# Synthetic hydrogel nanoparticles for sepsis therapy

**DOI:** 10.1038/s41467-021-25847-2

**Published:** 2021-09-21

**Authors:** Hiroyuki Koide, Anna Okishima, Yu Hoshino, Yuri Kamon, Keiichi Yoshimatsu, Kazuhiro Saito, Ikumi Yamauchi, Saki Ariizumi, Yuqi Zhou, Ting-Hui Xiao, Keisuke Goda, Naoto Oku, Tomohiro Asai, Kenneth J. Shea

**Affiliations:** 1 Department of Medical Biochemistry, University of Shizuoka, Shizuoka, 422-8526 Japan; 2 Department of Chemical Engineering, Kyushu University, Fukuoka, 819-0395 Japan; 3 Department of Chemistry, University of California Irvine, Irvine, CA 92697 USA; 4 Department of Chemistry, The University of Tokyo, Tokyo, 113-0033 Japan; 5 Institute of Technological Sciences, Wuhan University, Hubei, 430072 P. R. China; 6 Department of Bioengineering, University of California, Los Angeles, CA 90095 USA

**Keywords:** Sepsis, Nanobiotechnology, Nanoparticles

## Abstract

Sepsis is a life-threatening condition caused by the extreme release of inflammatory mediators into the blood in response to infection (e.g., bacterial infection, COVID-19), resulting in the dysfunction of multiple organs. Currently, there is no direct treatment for sepsis. Here we report an abiotic hydrogel nanoparticle (HNP) as a potential therapeutic agent for late-stage sepsis. The HNP captures and neutralizes all variants of histones, a major inflammatory mediator released during sepsis. The highly optimized HNP has high capacity and long-term circulation capability for the selective sequestration and neutralization of histones. Intravenous injection of the HNP protects mice against a lethal dose of histones through the inhibition of platelet aggregation and migration into the lungs. In vivo administration in murine sepsis model mice results in near complete survival. These results establish the potential for synthetic, nonbiological polymer hydrogel sequestrants as a new intervention strategy for sepsis therapy and adds to our understanding of the importance of histones to this condition.

## Abstract (web summary)

Sepsis caused by the release of inflammatory mediators into the blood is a life threatening disease. Here, the authors report on the development of hydrogel nanoparticles for the capture and neutralisation of histones, major inflammatory mediators, and demonstrate sepsis treatment in a murine model.

## Introduction

Sepsis is a life-threatening extreme response of the body to infection (e.g., bacterial infection and COVID-19), leading to the dysfunction of multiple organs via the release of diverse inflammatory mediators into the blood^1^. Patients with sepsis exhibit various symptoms such as fever, systemic hypotension, decrease in urine volume, impaired blood coagulation, increase in vascular permeability, and loss of vascular smooth-muscle tone^2,3^. The mortality of sepsis has been reported to be higher than that of stroke and myocardial infarction^4^. In addition, it has been reported that sepsis is strongly associated with coronavirus disease 2019 (COVID-19) caused by severe acute respiratory syndrome coronavirus 2 (SARS-CoV-2)^5^. Multiple factors contribute to the pathology of sepsis. During sepsis, systemic activation of the immune response is driven by the continuous secretion of alarmins such as high mobility group box 1 (HMGB1), multiple inflammatory mediators (cytokines), and cationic toxic proteins (histones) from damaged host tissue^6^ into the bloodstream. The complexity of the released toxic agents can lead to lethal effects to the host and challenge the development of effective therapeutic interventions^7–9^.

Current sepsis therapy calls for symptomatic treatment (e.g., broad-spectrum antibiotics, intravenous fluids, and surgery), but there is no direct treatment for sepsis^3,10^. Now, neutralization of specific cytokines such as HMGB1^11^, IL-3^12^, and IL-6^13^ offer potential approaches for therapeutic intervention. A somewhat broader approach for direct sepsis treatment uses macrophage-like nanoparticles (NPs), consisting of a biodegradable polymeric NP core coated with cell membranes extracted from human macrophages to capture endotoxins such as lipopolysaccharide (LPS), IL-6, TNFα, and IFN-γ^14^. Although these approaches offer promise, each has limitations for example, it remains to be established if toxin sequestrants have sufficient capacity^15^ and if challenges in mass production, immunogenicity, and reproducibility can be brought under control. The fact that multiple factors are responsible for the pathology of sepsis, identifying the most important targets presents a strategic challenge to develop effective therapeutic interventions. Histones play an important role in the lethality of sepsis, but despite their importance, they have not received a great deal of attention as sepsis mediators. Targeting their sequestration and removal may provide an additional opportunity for direct treatment of sepsis^8^. Normally, histones are packed in cell nuclei but are released into extracellular space as a result of bacterial infection and massive inflammatory cytokine production^16^. Once released histones non-specifically bind to the cell membrane and trigger endothelial cell damage, organ failure, and death^8^. In addition, histones bind to platelets and induce coagulation, prolonging bleeding time by decreasing the number of platelets in the bloodstream. There are five major classes of histones (i.e., H1, H2A, H2B, H3, and H4), neutralization of histone H3 and H4, two arginine-rich subtypes of histones, have been shown to reduce the mortality of murine models of sepsis^8,17^. However, other histone subtypes including lysin-rich H1, H2a, and H2b have similar physiochemical properties^18^ and also induce inflammation in the body^8,19,20^. Therefore, broad and non-specific neutralization of all histone subtypes presents a novel opportunity for sepsis therapy.

**Fig. 1:**
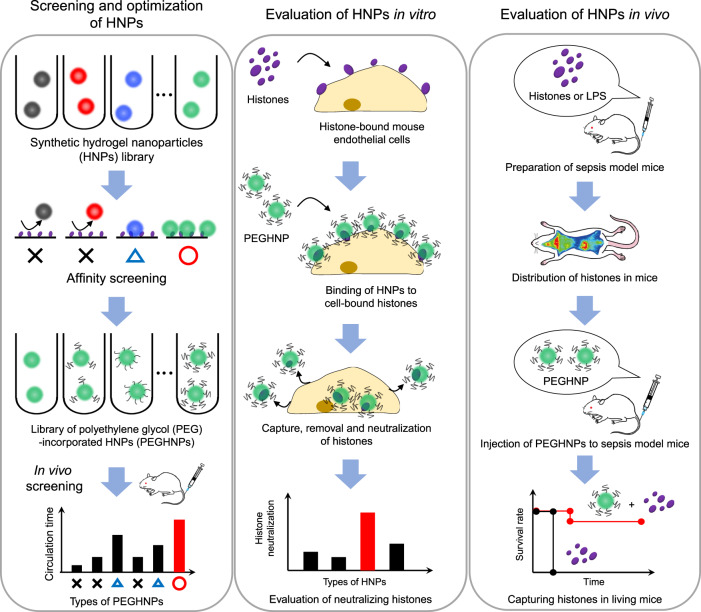
Workflow of this study. First, we develop HNPs with PEG modification to optimize their histone affinity, capacity, low-aggregation, and blood circulation time altogether to achieve highly effective sepsis therapy. Next, we evaluate PEGHNPs in vitro. Finally, we evaluate the efficacy of PEGHNPs using living mice in vivo.

In this article, we report the development of synthetic hydrogel NPs (HNPs) that neutralize all histone subtypes as a potential therapeutic agent for sepsis (Fig. 1). This work was motivated by prior studies that established the physiochemical properties of synthetic HNPs could be modified to tune both affinity and selectivity for specific biomacromolecule targets. Examples include affinity reagents for small molecules^21^, peptides^22–24^, enzymes^25^, polysaccharides^26^, lipid/lipid-like macromolecules^27^ and proteins^28,29^ including the angiogenesis signaling protein VEGF_165_^30,31^. The HNPs have a high capacity for target biomacromolecules compared with antibodies by sequestering them not only on their surface but also within the HNPs themselves^23^. These features notwithstanding, a current challenge in HNP-based therapy has been their short circulation time in the blood^23,31^. One attempt to improve these involved conjugating histone-capturing synthetic linear copolymers to a lipid NP (a highly biocompatible drug delivery agent), improving the bloodstream circulation time following intravenous injection^32^. However, these lipid NPs had a low capture capacity and tended to aggregate after histone collection.

In this work, to overcome these limitations, we designed and optimized an HNP with histone affinity, high capacity, low-aggregation tendency, and long blood circulation time to achieve a highly effective sepsis sequestrant. Specifically, we demonstrate that an optimized HNP captures and neutralizes histones over long periods of time. In addition, the HNPs showed extremely high binding capacity compared with antibodies, neutralized histones both before and after cell surface adhesion. Furthermore, HNP–histone complexes exhibited extremely low aggregation tendency. Most importantly, the HNPs significantly impacted survival in sepsis model mice by capturing and neutralizing resultant histones in the bloodstream. The abiotic HNPs hold significant promise as part of a direct treatment strategy for sepsis.

## Results

### Evaluation of the binding of synthetic HNPs to histones

**Fig. 2:**
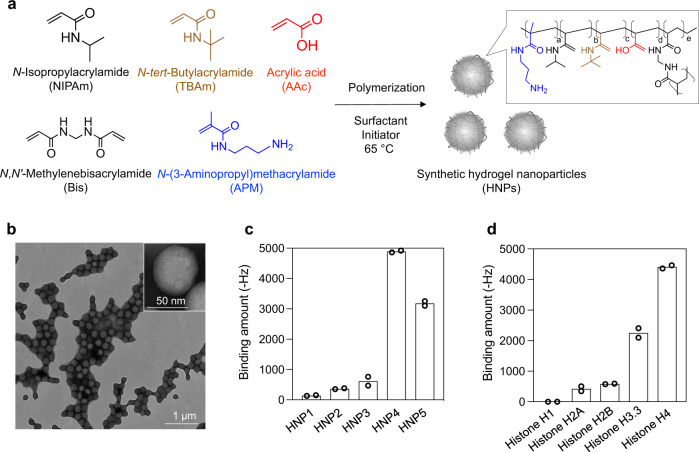
Screening of HNPs for histone affinity. **a** Schematic of synthetic HNPs synthesized by free-radical copolymerization of functional monomers. **b** TEM image of HNPs. The experiment was repeated three times. **c** Affinity of HNPs for histones. QCM sensor cells were functionalized with histones. After blocking with BSA, each type of HNPs was added and their binding amount was measured. **d** Affinity of HNP4 for the purified histone subtypes. QCM sensor cells were functionalized with the purified histone subtypes. After blocking with BSA, HNP4 was added and its binding amount was measured. Data represent the means of independent duplicate measurements.

Although there are five major classes of histones (i.e., H1, H2A, H2B, H3, and H4), all subtypes are positively charged proteins at physiological pH. To identify candidate NPs with high histone affinity, a small library of lightly (2%) cross-linked *N*-isopropylacrylamide-based hydrogel polymer NPs (HNPs) were prepared by a modified precipitation polymerization^33^. The HNPs incorporated combinations and permutations of functional monomers that include *N*-*tert*-butylacrylamide (TBAm, hydrophobic monomer), acrylic acid (AAc, negatively charged monomer), and/or *N*-(3-aminopropyl)-2-methylacrylamide hydrochloride (APM, positively charged monomer) (Fig. 2a). Since we previously reported that the inclusion of TBAm and AAc in the NIPAm-based HNP capture positively charged target peptides even in the bloodstream, we selected these functional monomers in this study. Monomer feed ratio, particle sizes, zeta-potentials, polydispersity index (PDI), and yield are summarized in Table 1. Transmission electron microscope (TEM) images and dynamic light scattering (DLS) indicated that the synthesized HNPs were mono-disperse (Fig. 2b). A quartz crystal microbalance (QCM) sensor functionalized with histones was used to evaluate the small HNP library for protein (histone) binding (Fig. 2c). This screening showed that the binding amount of HNP4 (AAc; 5%, TBAm; 40%) and HNP5 (AAc; 40%, TBAm; 40%) to histones was large. HNPs lacking a hydrophobic component (HNP1), containing positively charged monomers (HNP2), or those lacking any charged monomer (HNP3) showed little binding. Overall, the combination of hydrophobic and negatively charged groups was important to induce high histone binding amount. To determine the binding amount of HNP4 and HNP5 for each histone subtype, purified histone H1, H2A, H2B, H3, or H4-immobilized QCM sensor cells were used (Fig. 2d, Supplementary Fig. 1). Particularly, histones H3 and H4 are known to have strong toxicity^8,17^, and both HNP4 and HNP5 showed a large binding amount for these subtypes. Histones H3 and H4 have a significant amount of Arg in their sequence as well as high hydrophobic content (Supplementary Fig. 2). Arg can interact with carboxylic acids via a combination of electrostatic and hydrogen bonding interactions^34^, reinforcing the fact that HNP-histone binding requires HNPs with combinations of charged and hydrophobic groups. As histone H4 was reported to be the most critical sepsis mediator^8^, we chose HNP4 (which showed the largest binding amount for histone H4) for subsequent experiments. The apparent dissociation equilibrium constants (*K*_d_) of HNP4 for each purified histone ranged between 250 and 880 nM (Supplementary Fig. 3, Table 2). Noteworthy, the binding capacity of HNP4 for each purified histone (mg protein/mg HNP) ranged from 2 to 10. Significantly, HNP4 had an extremely high binding capacity for histones H3 and H4 (Table 2). The value compares favorably with antibodies since one antibody (M.W: 150 kDa) has the potential to capture only two target histones (M.W: ~10 kDa) which translates to a binding capacity of only (mg protein/mg antibody) ~0.14.

**Table 1:** Monomer composition, size, zeta-potential, and PDI of HNPs.

	NIPAm	TBAm	AAc	APM	Bis	Size (d.nm)	PDI	Zeta-potential (mV)	Yield (%)
HNP1	93	0	5	0	2	183 ± 19	0.07 ± 0.01	−22 ± 2	89 ± 4
HNP2	53	40	0	5	2	120 ± 13	0.12 ± 0.03	+30 ± 3	70 ± 4
HNP3	58	40	0	0	2	96 ± 1	0.02 ± 0.01	−39 ± 2	84 ± 3
HNP4	53	40	5	0	2	107 ± 3	0.02 ± 0.01	−35 ± 4	88 ± 2
HNP5	18	40	40	0	2	155 ± 20	0.04 ± 0.01	−40 ± 1	87 ± 3

The monomer structures can be found in Fig. 1a. Data represent the means ± s.d. *n* = 3.

**Table 2:** Binding affinity (*K*_d_) and capacity of HNP4 against purified histone subtype.

Histone	K_d_ (nM)	Protein (mg)/HNPs (mg)
H1	–	0
H2A	880	1.5
H2B	520	2.1
H3.3	250	5.6
H4	560	9.8

### Effect of PEG incorporation on in vivo HNP circulation time and histone affinity and capacity

**Fig. 3:**
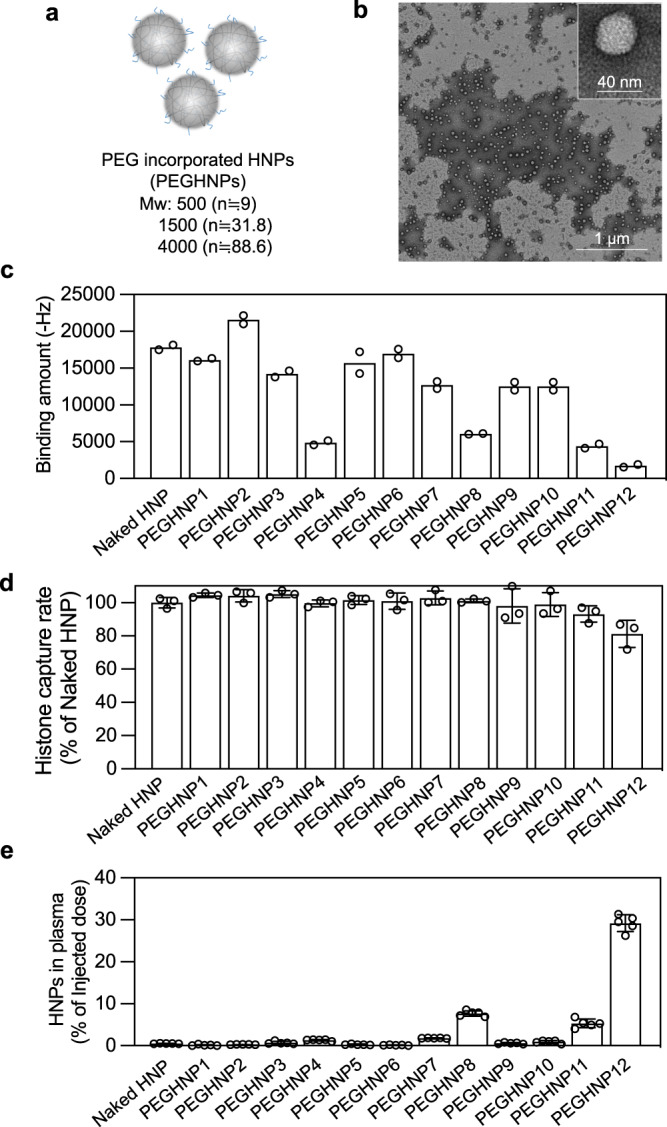
Incorporation of the optimum amount of PEG into HNPs for increasing the circulation time of HNPs without histone affinity reduction. **a** Schematic of PEGHNPs synthesized by free-radical copolymerization of functional monomers. **b** TEM image of PEGHNPs. The experiment was repeated two times, five pictures were taken in each experiment. **c** QCM analysis of the histone–PEGHNP interaction. The surface of the QCM cell was functionalized with histones and solutions of PEGHNPs were added to the QCM cells. Data represent the means of independent duplicate measurements. **d** Histone capture rate of PEGHNPs. Histone (600 µg/ml) and PEGHNPs (3000 µg/ml) were ultracentrifuged after the incubation for 30 min. Then, free histones were measured. Data represent the means ± s.d. *n* = 3. **e** Distribution of PEGHNPs in the plasma 3 h after the intravenous injection of [^14^C]-labeled PEGHNPs. Data represent the means ± s.d. *n* = 5. Blue bars; M.W of PEG: 500, yellow bar; M.W of PEG; 1500, and red bar; M.W of PEG; 4000.

Histones interact with cell surfaces by electrostatic interactions and can cause cell death under certain conditions within 10 min after in vitro addition^35^. Therefore, intercepting and clearing histones in the bloodstream as rapidly as possible is important for effective sepsis therapy. Most previous studies of poly(*N*-isopropylacrylamide) (pNIPAm)-based therapeutic HNPs found that they are rapidly cleared from the bloodstream shortly after intravenous injection^23,31^. Many of these applications were for the immediate removal of a toxin, circulation time was not a factor^23,36^. Improvement of HNP blood circulation time is best achieved by modification of the chemical composition of the HNP. Polyethylene glycol (PEG) modification of both biological and synthetic NPs is known to improve blood circulation time in vivo by reducing interaction with plasma proteins and opsonization^3^. However, since target affinity and selectivity are based on the HNP chemical composition, such modifications run the risk of loss of target affinity and selectivity. We hypothesized that although the incorporation of a large excess of PEG into the HNPs would likely reduce affinity for both plasma and histone proteins, it might be possible to fine-tune PEG length and amount to optimize circulation time without serious reductions to affinity and capacity. In the event, PEG methacrylate lengths (M.W: 500, 1500 or 4000 g/mol) were added to the initial monomer solution at feed ratios of 0.1, 0.3, 1, or 3 mol%, resulting in synthesized PEG-incorporated HNPs (PEGHNPs, Fig. 3a, Supplementary Fig. 4). TEM images show monodisperse synthetic PEGHNPs (Fig. 3b). Particle sizes and zeta-potentials of PEGHNPs are summarized in (Table 3). Incorporation of PEG in the synthetic NPs was confirmed by solution NMR which established a linear relationship between the PEG methacrylate feed ratio and the final incorporation ratio (Supplementary Figs. 5 and 6, Supplementary Table 1). An increase of PEG molecular weight and mole percentage in the HNPs resulted in decreased size and increased transparency of the HNP solution (Supplementary Fig. 7).

**Table 3:** PEG M.W., size, zeta-potential, and PDI of PEGHNPs.

	PEG M.W.	Feed ratio (mol%)	Size (d.nm)	PDI	Zeta potential (mV)	Yield (%)
PEGHNP1	500	0.1	149 ± 8	0.04 ± 0.02	−34 ± 5	84 ± 2
PEGHNP2	500	0.3	159 ± 24	0.06 ± 0.06	−33 ± 6	83 ± 7
PEGHNP3	500	1	175 ± 40	0.02 ± 0.01	−35 ± 2	79 ± 10
PEGHNP4	500	3	243 ± 37	0.08 ± 0.06	−29 ± 2	60 ± 3
PEGHNP5	1500	0.1	149 ± 26	0.04 ± 0.04	−34 ± 1	77 ± 3
PEGHNP6	1500	0.3	163 ± 24	0.04 ± 0.03	−33 ± 1	81 ± 3
PEGHNP7	1500	1	164 ± 14	0.04 ± 0.01	−30 ± 1	78 ± 6
PEGHNP8	1500	3	86 ± 2	0.04 ± 0.04	−18 ± 3	79 ± 2
PEGHNP9	4000	0.1	206 ± 49	0.04 ± 0.04	−26 ± 7	80 ± 5
PEGHNP10	4000	0.3	125 ± 4	0.05 ± 0.03	−26 ± 1	81 ± 5
PEGHNP11	4000	1	97 ± 2	0.07 ± 0.02	−20 ± 6	84 ± 13
PEGHNP12	4000	3	59 ± 3	0.14 ± 0.01	−14 ± 5	81 ± 17

Data represent the means ± s.d. *n* = 3.

It was reported that PEG chains locate mainly on the p(NIPAm) particle surface at temperatures above the lower critical solution temperature^37^. All our studies are run above the LCST of the HNPs. To produce evidence that PEG chains are exposed on the HNP surface, PEGHNPs were added to histone-immobilized QCM cells (Fig. 3c, Supplementary Fig. 8a). Comparing these assays with those using non-PEGlyated HNPs, the binding amount of PEGHNPs with histone-immobilized QCM cells *decreased* on average with increasing PEG molecular weight and incorporation amount. The reduced binding amount of PEGylated HNPs to the histone-immobilized QCM cells supports the notion that PEG chains accumulate on the surface. However, the study did not address the question of histone capacity by the PEGylated HNPs since histones must penetrate the PEG chains and bind to the HNP core. If histones still bind to the HNP core, the binding affinity and capacity of PEGHNPs for histones may not significantly decrease compared with non-PEGylated HNPs (naked HNP). To explore this point, the dissociation equilibrium constant (*K*_d_) of PEGHNPs for histones was measured by adding histones to HNP-immobilized QCM cells (Supplementary Figs. 8b, 9, Supplementary Table 2). Interestingly, the *K*_d_ of PEGHNPs for histones *decreased* upon PEG incorporation. *K*_d_ of PEGHNP12 was more than three times smaller than that of naked HNPs. The origins for this are no doubt complicated but it is known that the PEG structure has an intrinsic affinity to positively charged substrates^38^, which could be a contributing factor to increased affinity to histones. To summarize, the judicious introduction of PEG groups increased histone affinity. To identify the binding capacity of HNPs for histones, HNPs (3 mg/ml) and histones (600 µg/ml) were incubated and ultra-centrifuged (Fig. 3d). The binding capacity for histones decreased somewhat in PEGHNP11, 12 (~18% decrease) compared with naked HNP, however, PEGHNPs still had a very high binding capacity for histones (mg protein/mg HNP = 2–8). compared with antibodies (mg protein/mg antibody = ~0.14).

We then moved to demonstrate the effect of HNP PEGylation on blood circulation time and biodistribution. These parameters were measured at 3 and 24 h following intravenous injection of ^14^C-labeled PEGHNPs into the BALB/c male mice (Fig. 3e, Supplementary Figs. 10 and 11). Naked HNPs had a short residence time in the bloodstream (with ~0.5% remaining) and accumulated mainly in the liver (~50%) and kidneys (~10%) 3 h after i.v. injection. Although the biodistribution of PEGHNP1–7, 9, 10 was not significantly different from naked HNPs, PEGHNP8, 11, and 12 remained in the blood longer (~7.6%, ~5.3%, and ~30.4% remaining, respectively (Fig. 3f, Supplementary Fig. 10). At 24 h after i.v. injection, ~11% PEGHNP12 and ~3% PEGHNP8, 11 still remained in the bloodstream, however, only 0.07% of naked HNPs remained (Supplementary Fig. 11). The remaining PEGHNP8, 11 in the bloodstream was more than 30 times higher and PEGHNP12 more than 150 times higher than that of naked HNPs. The results establish that PEG incorporation significantly increases the HNP circulation time without dramatically affecting the binding affinity and sequestration capacity of HNPs for target histone proteins. This observation mirrors similar findings of PEGylation of therapeutic proteins^39^. Based on this result, the longer circulating PEGHNP8, 11 and 12 were chosen for the subsequent in vivo experiments.

### Neutralizing histones by HNPs after their cell adhesion

**Fig. 4:**
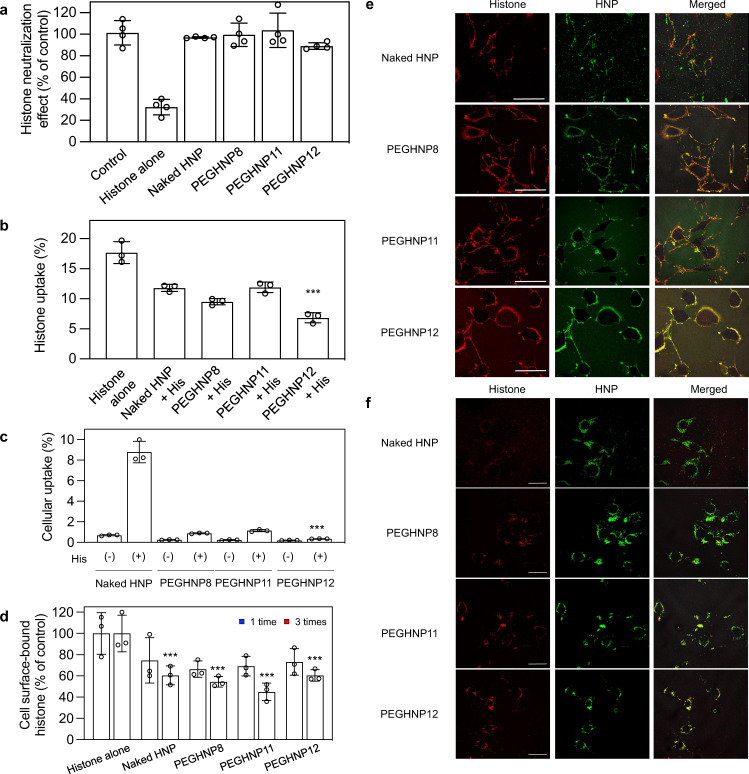
Neutralization of histones by HNPs after their binding to the cell surface. **a** Neutralization of the cell surface-bound histones by PEGHNPs. 2H-11 cells were treated with histones for 60 s. Then, PEGHNPs were added after the PBS washing to remove free histones in the medium. At 24 h after HNP addition, viable cells were determined by a WST-8 assay. Data represent the means ± s.d. *n* = 4. **b** Cellular uptake of histones. 2H-11 cells were incubated with Cy5-histones and PEGHNPs for 24 h. Then, the cells were lysed and the amount of Cy5-histones was measured. Significant difference; ***p < 0.0001 vs. Histones alone, *p* = 0.0012 vs. Naked HNP + His, *p* = 0.062 vs. PEGHNP8 + His, and *p* = 0.0001 vs. and PEGHNP11 + His. Data represent the means ± s.d. *n* = 3. Differences within a group were evaluated by one-way analysis of variance (ANOVA) with the Tukey post hoc test using Kaleidagraph (Version 4.5.3). **c** Cellular uptake of HNPs. 2H-11 cells were incubated with FITC-HNPs and/or histones for 24 h. Then, the cells were lysed and the amount of FITC-HNPs was measured. Significant difference; ***p < 0.0001 vs. Naked HNP(+), *p* = 0.0001 vs. PEGHNP8 (+), and *p* < 0.00012 vs. PEGHNP11 (+). Data represent the means ± s.d. *n* = 4. The data were analyzed by two-tailed Student’s *t* tests. **d** Removal of the cell surface-bound histones by PEGHNP washing. 2H-11 cells were treated with Cy5-labeled histones for 10 s. The cells were washed with PEGHNPs 1 or 3 times. Then, the cells were lysed and the fluorescent intensity of Cy5-histones was measured. Significant difference; *p* = 0.046 Histone alone vs. Naked HNP, *p* = 0.046 Histone alone vs. PEGHNP8, *p* = 0.046 Histone alone vs. PEGHNP11, and *p* = 0.026 Histone alone vs. PEGHNP12. Data represent the means ± s.d. *n* = 3. The data were analyzed by two-tailed Student’s *t* tests. **e**, **f** Localization of Cy5-histones and FITC-PEGHNPs. 2H-11 cells were treated with Cy5-histones for 10 s. Then, the cells were washed with FITC-PEGHNPs. At 30 min (**e**) and 24 h (**f**) after the washing, the localization of Cy5-histones and FITC-HNPs was observed laser scanning confocal microscopy. Red: histones; Green: HNPs; Bar: 50 µm. The experiment was repeated two times and three pictures were taken in each experiment.

Once histones are introduced into the bloodstream, they interact with endothelial cells^9^. Histones bind to cell membrane phospholipids and exhibit lethal toxicity such as endothelial dysfunction. In the presence of histone sequestering PEGHNPs, histones will be partitioned between the bloodstream and cell surfaces. To establish if HNPs neutralize histone toxicity after cell surface capture, pre-incubated HNPs (20 µg/mL) and histones (45 µg/mL) were added to 2H-11 mouse endothelial cells. Then, viable cells were determined by a WST-8 assay. The histone toxicity was dose-dependently inhibited by naked HNPs (without PEG, Supplementary Fig. 12). Noteworthy, all PEGHNPs also inhibited toxicity (Supplementary Fig. 13) indicating both HNPs (PEGHNPs and naked HNPs) were effective at neutralizing histones under these conditions. Subsequently, we evaluated whether HNPs neutralize histones after binding to cells. 2H-11 cells were incubated with histones for 1 min, then, after removal of histones in the culture medium by washing the cells 3 times with PBS, HNPs were added to the cells (Fig. 4a). Histone toxicity was found to be dose-dependently inhibited by HNPs, indicating HNPs could neutralize histones even after binding to cells. Importantly, both naked HNPs and PEGHNPs alone did not show in vitro cytotoxicity themselves at an HNP concentration of 300 µg/mL (Supplementary Fig. 14a). Histones on the other hand are cytotoxic at concentrations as low as 30 µg/ml (Supplementary Fig. 14b). Both HNPs and histones are taken up in cells but at significantly different rates. Fluorescence labeling studies showed HNPs alone had little cellular uptake. To demonstrate cellular uptake of HNP–histone complexes, pre-mixed Cy5-conjugated histones (Cy5-histones) and FITC-labeled HNPs (FITC-HNPs) were added to 2H-11cells and incubated for 24 h. A fluorescence assay established cellular uptake of histones significantly *decreased* upon precomplexation with either naked HNPs or PEGHNPs (Fig. 4b). On the other hand, although cellular uptake of naked HNPs and PEGHNPs (without histones) were below 1% (naked HNPs; 0.6%, PEGHNPs; ~0.2%), naked HNP uptake increased 15-fold (~9%) upon histone complexation (Fig. 4c). However, cellular uptake of PEGHNPs amounted to only 0.3% upon histone complexation. These results indicate that naked HNP surface-bound histones enhanced cellular uptake of naked HNPs, however, histones sequestered by PEGHNPs inhibit their interaction with cell surfaces and cellular uptake.

### Evaluation of the effect of neutralizing histones with HNPs

We further investigated how HNPs neutralize cell surface-bound histones. 2H-11 cells were incubated with Cy5-histones (100 µg/ml) for 10 s. Then, the cells were washed with HNPs (400 µg/ml) twice (Fig. 4d). Interestingly, the amounts of cell-bound Cy5-histones both decreased with naked HNPs and PEGHNP8, 11, 12 washing compared with just simple PBS washing, indicating both HNPs removed ~55% of the Cy5-histones from the cell surface. Since PEGHNPs circulate for longer periods of time, the results suggest that circulating PEGHNPs can be significantly more effective by binding and removing cell surface-bound histones during the time they are circulating in the bloodstream. To understand the fate of the remaining cell-bound Cy5-histones after HNP washing, a new group of cells was washed with FITC-HNPs (naked HNP and PEGHNP8, 11, 12) twice after incubating cells with Cy5-histones for 10 s. Then, the localization of Cy5-histones and FITC-HNPs was observed with confocal laser scanning microscopy. FITC-HNPs (naked HNP and PEGHNP8, 11, 12) were co-localized with Cy5-histones on the cell surface at 30 min after HNP addition (Fig. 4e). On the other hand, FITC-HNPs were co-localized with Cy5-histones in the cells at 24 h after the addition (Fig. 4f). These results indicate that HNPs bind to cell surface-bound histones. Approximately, half were removed from the surface with HNPs and the other half of the HNP–histone complex remained on the cell surface and internalized into the cell. To understand the fate of the remaining HNP-histone complexes on the cell surface, localization of FITC-HNPs (naked HNP, PEGHNP12) and Cy5-histone were monitored by confocal laser scanning microscopy for 10 h after adding to the cells. (Supplementary Fig. 15). Cy5-histone accumulation in the cell increased time-dependently over 10 h in the HNP un-treated or naked HNPs-treated cells. Although Cy5-histone accumulation in the cell also increased time-dependently upon PEGHNP12 treatment over 6 h as quickly as naked HNPs, the cy5-histone accumulation within the cell gradually decreased after 6 h. We suggest that the positive charge of histones captured by PEGHNP12 is masked to some extent, reducing their interaction with nuclei and other cytosolic proteins and leading to their excretion from the cell. These results indicate that the HNP-histone complexes that remain on the cell surface are taken up into cells via cellular membrane penetration^40^ (Fig. 4f). Once inside the cells, HNP-borne histones are apparently not released into the cytosol and do not induce toxicity. Based on these results, we concluded that in the presence of HNPs, histone toxicity was neutralized by HNPs’ sequestration and removal of histones from the cell surface and also by HNPs’ complexation and internalization with histones, although the latter process amounted to only a small percentage of histone distribution on the cell surface.

### Selective binding of HNPs to histones in serum

Since histones are released in the bloodstream during sepsis, a sequestrant must function selectively in a complex biological milieu. The specificity of PEGHNP12 was demonstrated by incubating PEGHNP12 in 50% plasma (25 mg/ml protein concentration) or 50% plasma (25 mg/ml) containing histones (6 mg/ml) for 1 h at 37 °C. Then, HNPs were purified by gel filtration chromatography, and protein quantities in the HNP fraction (fraction Nos. 22–27) were measured by BCA assay (Supplementary Fig. 16). Only small amounts of proteins were detected in the HNP fraction after incubation of PEGHNP12 in 50% plasma. However, large amounts of proteins that included histones were detected in the HNP fraction after incubation of PEGHNP12 in 50% plasma spiked with histones. These results establish that PEGHNP12 absorbs relatively few plasma proteins and would have the capacity, therefore, to selectively capture histones in the bloodstream. It was reported that some formulations of hydrophobic polymer HNPs interact with plasma proteins such as albumin and fibrinogen^41^. Since PEGHNP12 contains both negatively charged (AAc) and hydrophobic functional groups, this combination or balance of functional groups results in a PEGHNP12 that is stable in plasma with no strong affinity for common, abundant proteins in plasma including albumin and fibrinogen^41^.

### Stability of HNPs in serum

An effective therapeutic must not only sequester histones but the histone–HNP complex must also remain soluble in the bloodstream. Aggregation of HNP-histone complexes in the bloodstream could result in lethal side effects by blocking blood flow. Naked HNPs or PEGHNP12 were incubated with histones (100 mg/ml) or 50% plasma solution and the HNP size was measured by DLS (Supplementary Table 3). Neither naked HNP nor PEGHNP12 changed size after incubation in 50% plasma solution, however, only PEGHNP12 maintained its initial size (~50 nm) in the presence of histones. In addition, TEM images of PEGHNP12 after incubation with histones (100 mg/ml) for 1 h at 37 °C indicated the absence of aggregation (no particle size change) following incubation of PEGHNPs and histones (Supplementary Fig. 17), indicating that PEG-incorporation inhibits aggregation and PEGHNP12-histone complexes are stable in the bloodstream.

### Capturing histones by HNPs in the bloodstream of living mice

**Fig. 5:**
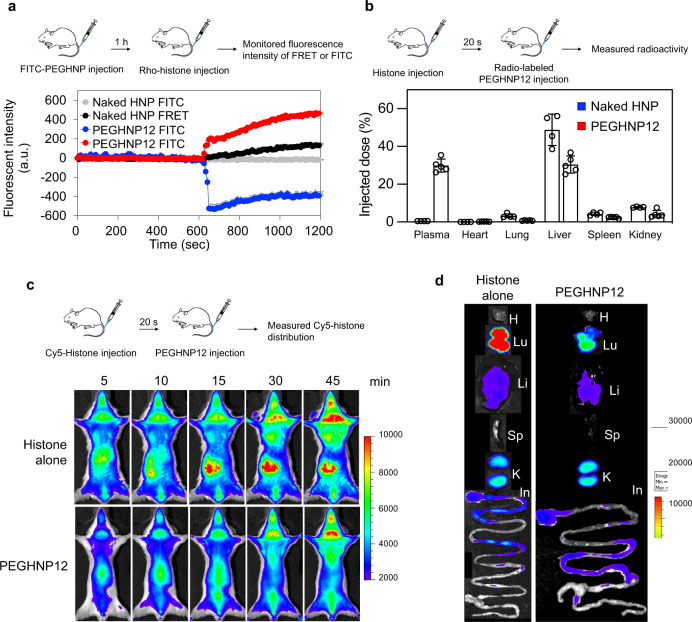
Capturing histones by HNPs in the bloodstream. **a** Behavior of Rho–histones and FITC–PEGHNPs in the murine bloodstream was observed by laser-scanning confocal microscopy. The FITC (Ex = 488 nm, Em = 520 nm) and FRET (Ex = 488 nm, Em = 570 nm) channels were recorded for 10 min before and after the histone injection. The fluorescence intensity of FRET or FITC in the bloodstream. The zero-time point corresponds to the time of the histone injection. Data represent the means ± s.d. *n* = 4 Rho; rhodamine, FITC fluorescein isothiocyanate. **b** Biodistribution of naked HNPs and PEGHNP12 in histone pre-treated mice. Mice were intravenously injected with radio-labeled naked HNPs or PEGHNP12 at 20 s after the histone treatment (IV). Twenty-four-hour after the injection, the distribution of HNPs in the plasma and each organ was measured. Data represent the means ± s.d. *n* = 5. **c**, **d** Biodistribution of Cy5-histones. Mice were intravenously injected with Cy5-histone after PEGHNP12 injection. Then, the biodistribution of cy5-histones were monitored by in vivo imaging system. **d** Ex vivo image at 1 h after the histone injection. H heart, Lu lungs, Li liver, Sp spleen, K kidneys, In intestine.

We next demonstrated whether HNPs capture histones in the bloodstream of living mice. Rhodamine-conjugated histones (Rho-histones), when complexed with FITC-labeled PEGylated (PEGHNP12) and non-PEGylated HNPs (naked HNPs), will undergo fluorescence resonance energy transfer (FRET) at 570 nm^42^. This diagnostic was used to detect Rho–histone–FITC–HNP complexes in vivo. Rho–histones were intravenously injected into mice 1 h after i.v injection of FITC-HNPs. The bloodstream was monitored at FITC (HNP) (Ex = 488 nm, Em = 520 nm) and FRET (HNP–histone complex) channels (Ex = 488 nm, Em = 570 nm) by confocal laser scanning microscopy (Fig. 5a, Supplementary Fig. 18). Monitoring FITC and FRET signals began 30 min after HNP injection. Neither FITC nor FRET signals were detected before or after the Rho–histone injection in the FITC-labeled naked HNP treated mice. This result was expected from previous results that showed naked HNPs of similar composition is rapidly cleared from the bloodstream after intravenous injection (only a few % remaining 30 min after the injection^35^). On the other hand, strong FITC signals in the bloodstream were detected at 1 h after the FITC–PEGHNP12 injection. Importantly the FITC signals were reduced and FRET signals increased following the Rho-histone injection. These results are consistent with the conclusion that PEGHNP12 remains active in the bloodstream after circulating for 1 h.

We next evaluated the change in biodistribution of HNPs and histones following their introduction in the bloodstream. To establish the biodistribution change of HNPs, ^14^C-labeled HNPs were injected into the BALB/c male mice 20 s after intravenous injection of a lethal dose of histones (Fig. 5b). Three h after the histone injection, HNP radioactivity in plasma and each organ were measured. Naked HNP accumulation in the lung increased 10 times following histone injection compared to the accumulation of naked HNP alone in the absence of histone (Supplementary Fig. 10). The results suggest that naked HNPs bind histones and form larger aggregates in the bloodstream that eventually accumulate in the lung (first-pass organ after the tail vein injection). However, under similar conditions, the biodistribution of PEGHNP12 was similar to that of ^14^C-labeled PEGHNP12 in the absence of histones (Fig. 5b, Supplementary Fig. 10), indicating PEGHNP12 does not aggregate in the bloodstream after histone capture. Next, to evaluate change in histone biodistribution, Cy5-histones (50 mg/kg) were intravenously injected into the BALB/c male mice. Then, naked HNPs (10 mg/kg) or PEGHNP12 were intravenously injected 20 s after histone injection and the biodistribution of Cy5-histones was monitored with in vivo imaging systems (Fig. 5c, d). Strong Cy5-histone signals were observed in lung, kidney, and intestine after treatment of mice with Cy-histone. On the other hand, the Cy-histone signal in lung, kidney, and intestine was significantly diminished upon PEGHNP12 treatment compared with injection of Cy5-histone alone, indicating PEGHNP12 captured histones in the bloodstream and inhibited their accumulation in the lung, kidney, and intestine. These biodistribution studies give support to the conclusion that PEGHNP12 not only captures histones in the bloodstream but also significantly alters their biodistribution.

### Inhibition of the migration of platelets into lungs by HNPs in vivo

**Fig. 6:**
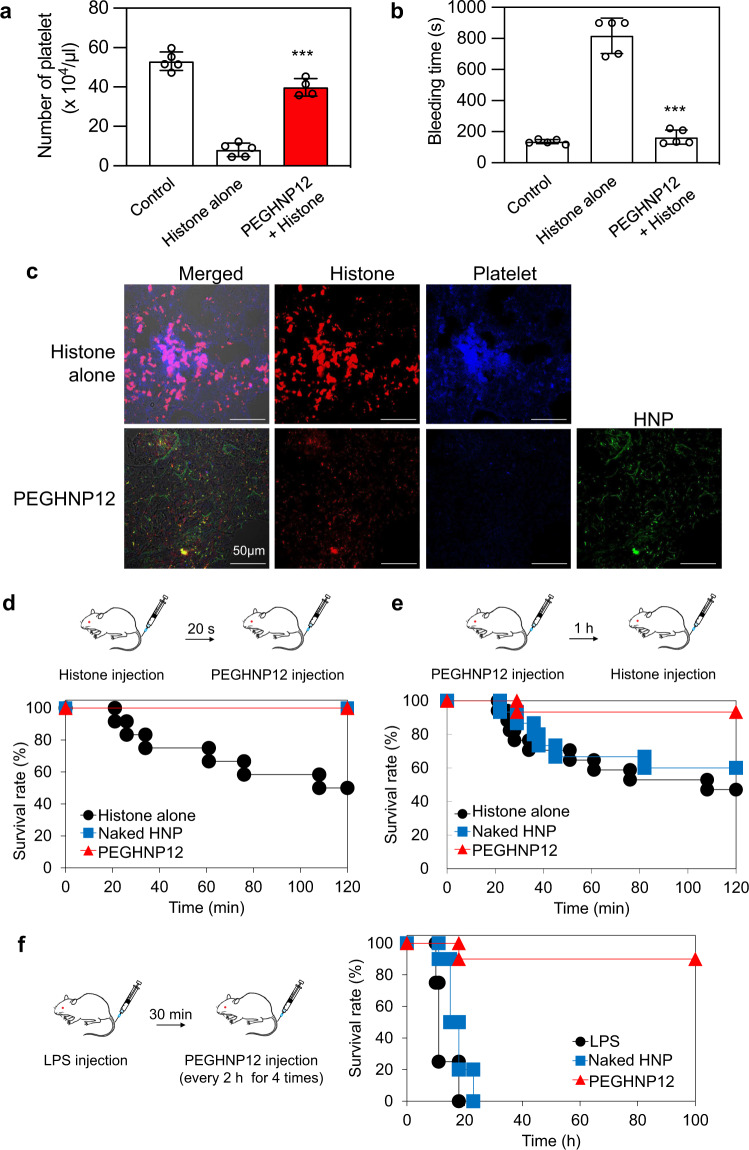
Protection of mice from septic lethality by HNPs in vivo. **a** Number of platelets after injecting histones and/or HNPs. Histones (40 mg/kg) were injected into mice 1 h after injecting HNPs (10 mg/kg). Twenty minutes after the injection, the number of platelets was measured. Significant difference; ***p < 0.0001 vs. Histones alone. Data represent the means ± s.d. *n* = 4–5. Data represent the means ± s.d. *n* = 4. Differences within a group were evaluated by one-way ANOVA with the Tukey post hoc test. **b** Tail bleeding time after injecting histones and/or PEGHNPs. Histones (40 mg/kg) were injected into mice 1 h after injecting HNPs (10 mg/kg). Twenty minutes after the injection, the time of bleeding from the tail was measured. Significant difference; ***p < 0.0001 vs. Histones alone. Data represent the means ± s.d. *n* = 4–5. Data represent the means ± s.d. *n* = 4. Differences within a group were evaluated by one-way ANOVA with the Tukey post hoc test. **c** Immunostaining of lungs after injecting histones and/or HNPs. Cy5-histones (40 mg/kg) were intravenously injected into mice 1 h after FITC-PEGHNP injection (10 mg/kg). Twenty minutes after the histone injection, the frozen section of the lungs was stained with CD41. Red: histones; blue: platelets; green: HNPs. Bar: 0.05 mm. **d** Survival rate of mice intravenously treated with PEGHNPs (10 mg/kg) at 20 s after the injection of histones (75 mg/kg). **e** Survival rate of mice intravenously treated with histones (75 mg/kg) at 1 h after the injection of PEGHNPs (10 mg/kg). **f** Survival rate of LPS-induced (15 mg/kg) sepsis model mice improved by the injection of PEGHNP12 (10 mg/kg). LPS lipopolysaccharide.

It was reported that histones bind to platelets in the bloodstream and induce aggregation and coagulation, resulting in decreasing the number of platelets and prolonging bleeding time^43^. To demonstrate inhibition of histone and platelet interaction by HNPs, FITC-PEGHNP12 (1 mg/ml), and Cy5-histones (1 mg/ml) were added to platelets (1 × 10^7^ cells). Although Cy5-histones adhered to and aggregated with platelets (Supplementary Fig. 19a), PEGHNP12 inhibited the interaction of Cy5-histones and platelets by capturing histones (Supplementary Fig. 19b). We next evaluated the number of platelets in blood after the intravenous injection of histones. Mice were intravenously injected with histones (40 mg/kg) 1 h after the PEGHNP12 injection (10 mg/kg). Then, the number of platelets in the blood and tail bleeding time was determined 20 min after the histone injection (Fig. 6a, b). Histone injection significantly reduced the number of platelets in the blood and prolonged tail bleeding time. However, pretreatment with PEGHNP12 significantly improved the number of platelets in the blood and inhibited prolonging tail bleeding time after histone injection. We found that intravenously injected histones accumulated in the lung (Fig. 6d) and make thrombi with platelets in the lung^43^. In fact, many platelets merged with histones were observed in the lung after the intravenous injection of Cy5-histones (40 mg/kg, Fig. 6c). In contrast to this, pretreatment with PEGHNP12 (10 mg/kg) inhibited migration of platelets into the lung, and histones within the lung were co-localized with PEGHNP12. These results indicate that PEGHNP12 effectively neutralized histones in the bloodstream and inhibited platelet activation, thrombus formation, and inflammation.

### Protection of mice from septic lethality by HNPs in vivo

Intravenous injection of histones induces septic characteristics such as neutrophil margination and accumulation in the alveolar microvessels, eventually causing death^8^. A standard sepsis mouse model was prepared by challenging it with a lethal dose of histones^8^. We used non-PEGylated HNP12 as a control to demonstrate the effect of PEG incorporation on in vivo histone neutralization ability. To study the efficacy of HNPs to neutralize histones in mice, HNPs (10 mg/kg) were intravenously injected into mice 20 s after intravenous injection of histones (75 mg/kg). PEGHNP12 was used for these in vivo studies. HNP treatment saved all mice (100% survival) from histone toxicity (Fig. 6d). In contrast, 50% of mice died following injection of histone alone, indicating a follow-on introduction of HNPs neutralizes histones when a lethal dose is introduced into the bloodstream of living mice. Since histones are continuously released during sepsis, sustained efficacy is influenced by long circulation times in the bloodstream. However, the long circulation of HNPs in the bloodstream increases the opportunity for interacting with plasma proteins with the potential to form a protein corona^41^. Protein corona formation would result in reduced affinity and capacity of HNPs for histones^44^. Therefore, increasing HNP circulation time in the bloodstream without reduction of histone capture ability is important for HNP efficacy. For this study, we compared the histone neutralizing effect of circulating PEGylated (PEGHNP12) and non-PEGylated HNPs (naked HNPs). Histones were intravenously injected into BALB/c male mice at 1 h after the intravenous injection of HNPs (Fig. 6e). Naked HNPs did not increase the survival rate of mice after histone injection compared with the control (PBS) group. In contrast, circulating PEGHNP12 significantly improved (2 times) the survival rate of mice that were intravenously injected with histones. PEGHNP12 also had a longer circulating time and showed little tendency to form complexes with plasma proteins. The results indicate that PEGHNP12 maintains a histone neutralizing capacity even after circulating in the bloodstream for 1 h.

Finally, the therapeutic effect of PEGHNP12 on sepsis was demonstrated using sepsis model mice induced by the intravenous injection of LPS^8^ (Fig. 6f). Importantly, we previously showed that TBAm containing HNPs have no binding for LPS^27^. It is known that injection of LPS into mice replicates the physiology of severe sepsis compared with just histone injection^8,45^. Since blood concentration of inflammatory cytokines such as tumor necrosis factor alfa and IL-10 dramatically increased at 1.5 h after the intravenous injection of LPS^46^, HNP should be injected within this time. PBS (control), naked HNPs, or PEGHNP12 were intravenously injected four times into the sepsis model mice every 30 min over a 2 h span following the LPS injection. All mice died within 20 h after the LPS injection followed by PBS or naked HNP treatment. However, under PEGHNP12 treatment significant improvement in survival (90%) was noted. These results establish that PEGHNP12 treatment can be effective for sepsis therapy by neutralizing histones both in the bloodstream and on the cell surface.

## Discussion

Although the number of deaths from sepsis has been increasing, no recent treatments have resulted in significant improvement in the survival rate. Since multiple factors contribute to the pathology of sepsis, identifying the most important targets presents a strategic challenge in the search for effective therapeutic interventions. Despite progress being made on multiple fronts to suppress the production or to sequester toxic factors that contribute to sepsis pathology, the number of deaths from sepsis has been increasing, no recent treatments have resulted in significant improvement in the survival rate. We have chosen to focus on histone sequestration. Histones are lethal basic proteins released during the later stages of sepsis. Here we demonstrate a strategy with the potential to impact the survival of patients with advanced-stage sepsis. The innovation is an abiotic synthetic copolymer, an HNP that neutralizes all histone subtypes in vivo. The HNP was formulated by optimizing the physicochemical properties by adjusting the anionic and hydrophobic monomer composition of the copolymer. Antibodies, synthetic polymers, and NPs designed with the ability to sequester toxins responsible for sepsis pathology such as macrophage membrane coated NPs^14^ or synthetic copolymers^32^ have also shown therapeutic effects for sepsis, validating this strategy as a potential therapeutic intervention. The success of these strategies is a function of many variables including their capacity for the target proteins which depends on the part surface area, a potential limitation to detoxification efficiency. The HNPs described in this report can capture toxins both on their surface and in their interior and have a significantly higher toxin capacity compared to particulate sequestrants such as antibodies and membrane-coated NPs. Furthermore, the optimized HNPs described in this report capture and neutralize significant quantities of all histone subtypes (mg protein/mg HNP = 2–10), a potential advantage over anti-histone antibodies (mg protein/mg antibody = ~0.14).

Although the physicochemical properties of HNPs can be altered to achieve both high affinity and selectivity^47^, these properties alone are insufficient for therapeutic efficacy; circulation time is also an important factor. Unfortunately, prior studies with HNPs have shown an inherently short circulation time^23,31^. Since affinity and selectivity depend on HNP chemical composition, a traditional method of enhancing circulation time by incorporating PEG chains presents a unique challenge. However, we have established in this report that incorporation of optimized length and amounts of PEG chains in the polymerization step resulted in HNPs with enhanced circulation time without significant reductions in target binding affinity and capacity. This allowed histones to be captured by HNPs across long time frames following intravenous injection. The characteristic of high binding capacity, broad-spectrum targeting, low aggregation, and long-circulating time of our HNPs should make them therapeutically relevant in the search for the treatment of severe inflammation and sepsis.

In its present form histone sequestration is subject to several limitations. First, although we have engineered HNPs for the efficient histone capture, other inflammatory cytokines such as high-mobility group box-1 (HMGB1) and TNFα also contribute to the sepsis pathology^48^. While inhibition of HMGB1^11^, selective cytokines^12^, and histones^8^ show a sepsis therapeutic effect, the shutdown of the cytokine storm and inflammation by neutralization of a significant fraction of all toxic and inflammatory agents will likely produce a more dramatic therapeutic response compared with single protein neutralization suggesting a combination therapy might be the most effective pathway. Second, although compositionally similar HNP’s were shown to be non-toxic and non-immunogenic^23,31^, HNPs might not be degraded by enzymes in the body, giving them the potential for long-term accumulation. Since multiple HNP injections are likely to be required for sepsis therapy, such accumulation may induce adverse downstream effects in the body. Therefore, a biodegradable characteristic could eventually be included in the HNP formulation should this become problematic. Third, in our study, we chose histone and LPS injection^49^ as murine sepsis models. There are however many murine sepsis models, some of which incorporate bacteria injection. A bacterial component was not addressed in this work but the therapeutic effect of HNPs should be explored using other sepsis model animals. This work will be pursued as this study advances.

The HNPs are amenable to alternative formats for histone sequestration. For example, preclinical results have been obtained with ex vivo blood purification therapies as adjuvant treatment for sepsis^50,51^, the HNPs described in this report may also serve in this capacity for the selective removal of histones from circulation. More broadly, abnormally high concentrations of molecules can be observed in several diseases such as cancer, diabetes, stroke, and COVID-19. It is theoretically possible to apply HNPs to these diseases by the optimization of functional monomer structure and amount against these targets. In our future efforts, we will explore HNP-based inhibition of sepsis-driven platelet aggregation using our previously reported image-based platelet aggregation classifier^52–54^. This study establishes the potential of non-biological synthetic polymer HNPs as selective protein affinity agents against intractable disease.

## Methods

### Materials

Histones from calf thymus, bovine serum albumin, *N*-isopropylacrylamide (NIPAm), sodium dodecyl sulfate (SDS), fluoresceinamine isomer, and poly(ethylene glycol) methyl ether methacrylate (average *M*_*n*_ 500, 1500, and 4000) were purchased from Sigma Aldrich. *N*,*N’*-methylenebisacrylamide (BIS) was from Fluka; *N*-t-butylacrylamide (TBAm) was from ACROS ORGANICS. [2,3-14C]-Acrylamide was purchased from GE Healthcare UK Ltd. (Buckinghamshire, UK). Histone H1, H2A, H2B, H3.3 and H4 (Human, Recombinant) were purchased from New England Biolabs, Inc., (MA, USA). Sulfo-Cy5 NHS ester was purchased from Lumiprobe (FL, USA). 5-(and 6)-carboxytetramethylrhodamine, succinimidyl ester (NHS-Rhodamine) was purchased from Thermo Fisher Scientific, Inc.

### Preparation of HNPs

HNPs were synthesized by free-radical copolymerization of *N-*isopropylacrylamide (NIPAm), *N,N’*-methylenebisacrylamide (Bis), *N*-t-butylacrylamide (TBAm), acrylic acid (AAc) and *N*-(3-aminopropyl)methacrylamide hydrochloride (APM). SDS (10 mg) were dissolved in water (50 mL). The solutions were filtered through a no. 2 Whatman filter paper (total monomer concentration; 65 mM). TBAm was dissolved in ethanol before the addition. Poly(ethylene glycol) methacrylates were added to the initial solution. Nitrogen was bubbled before the reaction for 30 min. Following the addition of ammonium persulfate aqueous solution (30 mg), the polymerization was carried out at 65 °C for 3 h under. The polymerized solutions were purified by dialysis.

### Characterization of HNPs

The hydrodynamic diameter of HNPs was determined by DLS (Zetasizer Nano ZS). The temperature of the HNP samples was controlled via the Peltier device at 25 ± 0.1 °C. Yield and concentration of HNPs were determined by measuring the weight of HNP after lyophilization of an aliquot.

### Quantification of functional monomers in HNPs by 1H-NMR

To determine the incorporation ratio of TBAm, NIPAm, and PEG in the polymer, ^1^H NMR spectroscopy was demonstrated (acquisition time; 30 s). NMR spectra were acquired in CD_3_OD and the chemical shifts are reported in ppm on the *δ* scale reference to residual CD_2_HOD (*δ* 3.31 ppm). The incorporation ratio of PEG and TBAm to NIPAm in the polymer was calculated from the integration of the methyl proton resonances at 1.36 (Tp), 1.16 ppm (Np), 3.63 ppm (Pp) using Eq. 1.

1 $${{{{{\rm{TBAm}}}}}}:{{{{{\rm{NIPAm}}}}}}:{{{{{\rm{PEG}}}}}}=({{{{{\rm{Tp}}}}}}/9):({{{{{\rm{Np}}}}}}/6):({{{{{\rm{Pp}}}}}}/4)$$

We used this relationship to approximate the incorporation of PEG in all of the HNPs synthesized in this study.

### Transmission electron microscopy image

HNPs (1 mg/ml) in a volume of 5 µL were placed on a grid (Nisshin EM, Tokyo, Japan) and dried with a stream of warm air 3 times. Then, each sample was negatively stained with 10 µL of 1 w/v% ammonium molybdate for 1 min and imaged with an HT7700 TEM System (Hitachi High-Technologies, Tokyo, Japan). The images were recorded with a CCD camera at 1024 × 1024 pixels (Advanced Microscopy Techniques, Woburn, MA, USA).

### QCM analysis

An Affinix Q^4^ and Q^8^ QCM instruments (Alvac Co. Ltd., Kanagawa, Japan) were used to quantify interactions between the HNPs and histones. Gold electrodes of the QCM cell were cleaned with piranha solution for 5 min, twice. 3,3′-Dithiodipropionic acid (1 mM, 0.1 mL) was added to the QCM cells and incubated overnight. Then, the QCM cells were washed with water and 1-ethyl-3-(3-dimethylaminopropyl)-carbodiimide (100 mg/ml, 50 µL) and *N*-hydroxysuccinimide (100 mg/mL, 50 µL) were added to the cell to form *N*-hydroxysuccinimidyl esters. Histones (30 µg/mL) were added onto the QCM gold surface (10 µL) and incubated for 1 h at 37 °C. The QCM cells were washed with water 2 times and blocked with 1 mg/mL BSA solution for 1 h. HNPs were added into the cells at concentrations of 100 µg/ml. Interactions between HNPs and proteins were observed at (37 ± 0.1) °C in PBS (pH 7.4).

### The apparent equilibrium dissociation constant (*K*_d_)

An Affinix Q^8^ QCM instrument (ULVAC Co. Ltd., Kanagawa, Japan) was used. Histones were immobilized in the QCM cells by amino coupling as described above. After washing the cells twice, HNPs were added into the cells until saturation. Then, the QCM cells were washed with PBS 2 times. Purified histone subtypes were added to the cell to measure the affinity of HNPs for the histone subtypes at (37 ± 0.1) °C in PBS (pH 7.4). The apparent dissociation constant of HNPs to protein was calculated under the assumption that all particles have the same average affinity to the protein.

### The binding capacity of HNPs for histone subtypes

The capacity of histones captured by HNPs was quantified by an Affinix Q^4^ QCM instrument. Histones were immobilized on the QCM cells by amino coupling as described above. After washing the cells twice, 800 ± 100 Hz of HNPs were immobilized on the surface. Then, the QCM cells were washed with PBS twice. Purified histone subtypes were added to measure the binding affinity and capacity. Frequency change was plotted against the concentration of histones and fitted to the Langmuir isotherm. The maximum absorption amount of histone was determined from the saturation point of the fitted curves. Binding capacity was determined by dividing the saturated amount of histone by the amount of immobilized HNPs. Here, the effect of water surrounding proteins and HNPs on the frequency change observed by the QCM has to be considered^55^. Thus, the effect of water on the frequency change was determined for histones and HNPs prior to the experiments as reported^55^.

### Histone capture rate by HNPs

Histone (600 µg/ml) and HNPs (3 mg/ml) were incubated for 30 min at 37 °C. Histones that were not captured by HNPs were measured by UV visible spectroscopy (280 nm) of the supernatant after ultracentrifugation (235,000× *g*, 37 °C, 30 min).

### Cell culture

2H-11 murine endothelial cells (ATCC, Virginia, USA) were cultured in DME/high-glucose medium (Wako Pure Chemical Industries, Ltd., Osaka, Japan) supplemented 10% FBS, 100 U/mL penicillin, and 100 μg/mL streptomycin at 37 °C in a humidified atmosphere of 5% CO_2_ in the air.

### Histone toxicity neutralization preincubation of HNPs and histones

2H-11 cells were seeded onto the 24-well plate at 6.0 × 10^3^ cells/well and incubated overnight. Then, the cells were treated with HNPs (20 µg/mL) and histones (45 µg/mL) after the preincubation of HNPs and histones for 30 min. Twenty-four h after the addition, the viable cells were determined by using a Cell Counting Kit-8 (Dojindo, Kumamoto, Japan) in accordance with the manufacturer’s instructions. For the measurement of HNP toxicity, cells were incubated with HNPs for 24 h and the viability was determined. Absorbance was measured with an Infinite^®^ M200 (Tecan Group, Männedorf, Switzerland) at a test wavelength of 450 nm and a reference wavelength of 630 nm.

### Neutralization of cell surface-bound histone toxicity

2H-11 cells were seeded onto the 24-well plate at 2.0 × 10^4^ cells/well and incubated overnight. The cells were treated with histones (500 µg/mL) for 1 min. Several concentrations of HNPs were added after washing the cells with PBS 3 times to remove histone in the medium. At 24 h after the histone addition, the viable cells were detected by using a Cell Counting Kit-8 in accordance with the manufacturer’s instructions.

### Preparation of sulfo-Cy5- or rhodamine-conjugated histones

Sulfo-Cy5 NHS ester or rhodamine NHS diluted with DMSO was incubated with histones (histones: sulfo-Cy5 NHS ester or rhodamine NHS = 1: 2 as a molar ratio) for 45 min at 25 °C. After the reaction, the solution was dialyzed against PBS for 2 days by use of a Float-A-Lyzer G2 Dialysis Device (MWCO: 3 kDa).

### Removal of cell surface-bound histones by HNPs washing

2H-11 cells were seeded onto the 24-well plate at 2.0 × 10^4^ cells/well and incubated overnight. The cells were treated with Cy5-histones (100 µg/mL) for 10 s. Then, the cells were washed with HNPs (400 µg/mL) or PBS 1 or 3 times. The cells were lysed with 1 w/v% noctyl-β-d-glucoside containing the following protease inhibitors: 1 mM phenylmethylsulfonyl fluoride, 2 μg/mL leupeptin, 2 μg/mL aprotinin, and 2 μg/mL pepstatin A. The fluorescence intensity of Cy5-histone was determined with a Tecan Infinite M200 microplate reader (ex. 640 nm, em. 670 nm).

### Localization of Cy5-histone and FITC-HNPs in vitro

2H-11 cells were seeded onto a glass-bottom 24-well plate (AGC Techno Glass Co., Ltd., Shizuoka, Japan) at 2.0 × 10^4^ cells/well and incubated overnight. The cells were treated with Cy5-histones (100 µg/mL) for 10 s. Then, FITC-HNPs (400 µg/mL) was added after washing the cells with PBS 3 times to remove histone in the medium. At 30 min and 24 h after the histone addition, the localization of Cy5-histone and FITC-HNPs was observed by confocal laser-scanning microscopy.

### Selectivity of HNPs to histones

PEGHNP12 were incubated in 50% plasma (25 mg/ml protein concentration) or 50% plasma (25 mg/ml) containing histones (6 mg/ml) for 1 h at 37 °C. Then, HNPs were purified by gel filtration chromatography and protein quantities in the HNP fraction (fraction Nos. 22–27, 1 mL in each fraction) were measured by BCA assay.

### Experimental animals

Five-week-old BALB/c male mice were purchased from Japan SLC Inc. (Shizuoka, Japan). All mice were male and housed in a temperature-controlled room (25 °C) with a 12-h light–dark cycle and 50 ± 10% humidity. They were allowed free access to food and water. The animals were cared for according to the Animal Facility Guidelines of the University of Shizuoka. All animal experiments were approved by the Animal and Ethics Review Committee of the University of Shizuoka. In addition, all experiments received ethical approval from the corresponding institute committee.

### Biodistribution of HNPs in mice

HNPs were radiolabeled by the inclusion of a small amount of [^14^C]-acrylamide. Mice were injected with [^14^C]-labeled HNPs (74 kBq/mouse [*n* = 6]) via a tail vein. For the measurement of the biodistribution of HNPs and histone complex, mice were intravenously injected with [^14^C]-labeled HNPs at 20 s after the intravenous injection of histones. Three or twenty-four hours after the administration, the mice were sacrificed under deep anesthesia with isoflurane for the collection of blood. Then, the blood was heparinized and separated by centrifugation (700 × *g*, 15 min, 4 °C) to obtain the plasma. After the mice had been bled from a carotid artery, their heart, lungs, liver, spleen, and kidneys were removed and weighed. The radioactivity in plasma and each organ was determined with a liquid scintillation counter (LSC-3100, Aloka, Tokyo, Japan). The total radioactivity in the plasma was calculated based on the average body weight of the mice, where the average plasma volume was assumed to be 4.27% of the bodyweight based on the data on total blood volume.

### Biodistribution of histone in mice

Mice were injected with HNP (10 mg/kg) via a tail vein 20 s after Cy5-histone (50 mg/kg) injection. Then, in vivo imaging was performed by in vivo imaging system. One hour after the histone injection, the mice were sacrificed under deep anesthesia with isoflurane. Then, ex vivo imaging was performed using the heart, lungs, liver, spleen, kidneys, and intestine. The living image software was used for data acquisition (Living Image 3.2, Xenogen Corp., Alameda, CA, USA)

### In vivo real-time imaging of FITC-HNPs and Rhodamine-histone

For the in vivo real-time imaging of HNP and histones in the bloodstream, mice were anesthetized with 2.5% isoflurane and the ear was set on the cover glass. The mice were intravenously injected with FITC-HNPs (600 µg/mouse). At 1 h after the injection, rhodamine–histone (180 µg/mouse) was intravenously injected into the mice. Bloodstream in-ear (every 8 s) was observed 10 min before and after the rhodamine-histone injection under an A1R + confocal laser-scanning microscope (Nikon, Tokyo, Japan). FITC channel: Ex = 488 nm, Em = 520 nm. FRET channel: Ex = 488 nm, Em = 570 nm.

### HNPs treatment after intravenous injection of histones

Mice were intravenously injected with histones at the concentration of 75 mg/kg. At 20 s after the injection, PBS or HNPs (10 mg/kg) was intravenously injected into the animals.

### HNPs treatment before intravenous injection of histones

Mice were intravenously injected with PBS or HNPs (10 mg/kg). One hour later, histone (75 mg/kg) was intravenously injected into the animals.

### Inhibition of platelets and histone interaction by HNPs

Blood was collected from the hearts with 0.38% citric acid (pH = 5.5) under deep anesthesia with isoflurane. The blood was centrifuged (80×*g*, 10 min, 4 °C) to collect platelet-rich plasma. Then, 1 volume of the platelet-rich plasma was added to 1 volume of Tyroad buffer containing prostaglandin E1 (5 µg/ml) and apyrase (2 U/ml) and centrifuged (400 × *g*, 5 min, 4 °C). The platelets were stained with CellTraceTM Violet (Invitrogen, Rockville, MD) according to the manufacturer’s instructions. Then, FITC-PEGHNP12 (1 mg/ml) and Cy5-histone (1 mg/ml) was added to the platelets (1 × 10^7^ cells). The platelets, Cy5-histone and FITC-HNPs were observed by confocal laser-scanning microscopy.

### Measurement of the number of platelets in the blood and bleeding time

Mice were intravenously injected with histones (40 mg/kg) 1 h after the PEGHNP12 injection (10 mg/kg). Twenty min after the histone injection, the blood was collected from the hearts with 0.38% citric acid (pH = 5.5) under deep anesthesia with isoflurane. Then, the number of platelets was measured with Celltac α (NIHON KOHDEN CORPORATION, Japan, Tokyo). For the measurement of bleeding time, 3 mm of the distal mouse tail was cut and immersing the tail in PBS (37 °C) 20 min after the histone injection. Then, bleeding time was measured. Importantly, measurement of bleeding time was stopped at 900 s.

### Localization of histone, platelets, and HNPs in the lung

Mice were intravenously injected with Cy5-histones (40 mg/kg) 1 h after the FITC-PEGHNP12 injection (10 mg/kg). Thirty min after the histone injection, frozen section of lungs (20 µm) were incubated with mouse integrin alpha 2b/CD41 Alexa fluor^®^ 594-conjugated antibody (USA, Minneapolis, MN, 25 times dilute for use) for 1 h at room temperature after the blocking with 1% BSA-PBS for 1 h at room temperature. Then, the sample was fixed with 4% paraformaldehyde for 30 min at room temperature. Localization of platelets, Cy5-histone, and FITC-HNPs were observed by confocal laser-scanning microscopy.

### The therapeutic effect of HNPs on sepsis model mice

Mice were intravenously injected with LPS at the concentration of 15 mg/kg. Thirty minutes later, PBS or HNPs (10 mg/kg) was intravenously injected into the mice 4 times every 2 h.

### Statistical analysis

Differences within a group were evaluated by analysis of variance (ANOVA) with the Tukey post hoc test and two-tailed Student’s *t* tests using Kaleidagraph (Version 4.5.3). Standard division (s.d was calculated by Prism 9 (Version 9.2.0)).

### Reporting summary

Further information on research design is available in the Nature Research Reporting Summary linked to this article.

## Supplementary information

Supplementary Information

Reporting Summary

## Data Availability

The data that support the findings in the present study are available from the corresponding author upon reasonable request.
