# Supplementary material for: Synthetic hydrogel nanoparticles for sepsis therapy
Source: Nat Commun. 2021 Sep 21;12:5552. doi: 10.1038/s41467-021-25847-2 (PMC8455629; doi:10.1038/s41467-021-25847-2)
Supplement: Supplementary file 1 — Supplementary Information [file 41467_2021_25847_MOESM1_ESM.pdf]

Supplementary Information

Synthetic hydrogel nanoparticles for sepsis therapy

Hiroyuki Koide[1*], Anna Okishima[1], Yu Hoshino[2*], Yuri Kamon[3], Keiichi Yoshimatsu[3], Kazuhiro Saito[1], Ikumi Yamauchi[1], Saki Ariizumi[1], Yuqi Zhou[4], Ting-Hui Xiao[4], Keisuke Goda[4,5,6], Naoto Oku[1], Tomohiro Asai[1], and Kenneth J. Shea[3*]

[1]*Department of Medical Biochemistry, University of Shizuoka, Shizuoka 422-8526, Japan.*
[2]*Department of Chemical Engineering, Kyushu University, Fukuoka 819-0395, Japan.*
[3]*Department of Chemistry, University of California Irvine, Irvine, California 92697, USA.*
[4]*Department of Chemistry, The University of Tokyo, Tokyo 113-0033, Japan.*
[5]*Institute of Technological Sciences, Wuhan University, Hubei 430072, P. R. China.*
[6]*Department of Bioengineering, University of California, Los Angeles, California 90095, USA.*

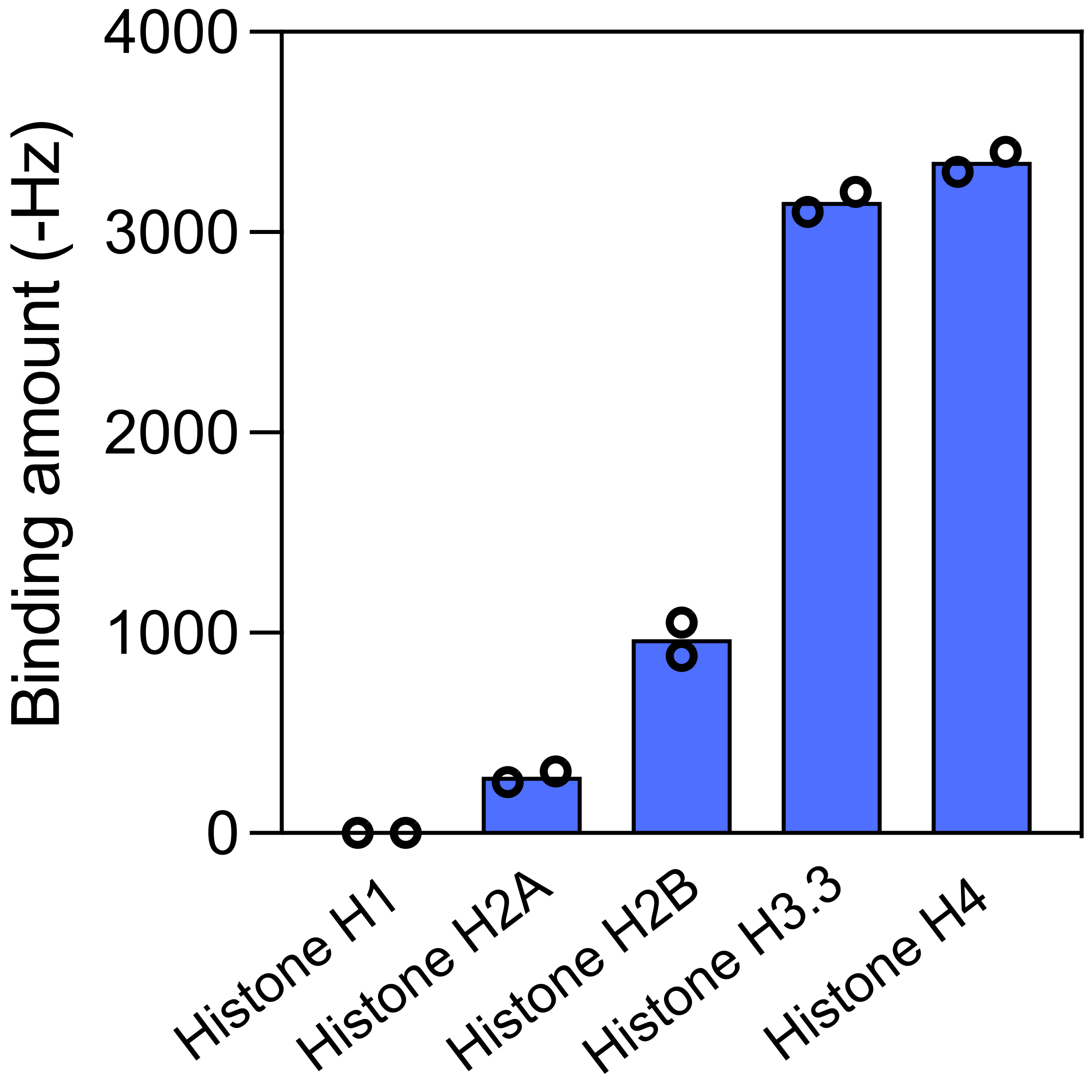

Supplementary Figure 1. QCM analysis of HNP5 – purified histone subtype interaction. The surface of the QCM was functionalized with purified histone and solutions of HNP5 was added to the QCM cells. Data represent the means for independent duplicate measurements.

・Histone H1
MSETVPPAPA ASAAPEKPLA GKKAKKPAKA AAASKKKPAG PSVSELIVQA ASSSKERGGV SLAALKKALA AAGYDVEKNN SRIKLGIKSL VSKGTLVQTK GTGASGSFKL NKKASSVETK PGASKVATKT KATGASKKLK KATGASKKSV KTPKKAKKPA ATRKSSKNPK KPKTVKPKKV AKSPAKAKAV KPKAAKARVT KPKTAKPKKA APKKK

・Histone H2A
MSGRGKQGGK ARAKAKSRSS RAGPQPPAGR AHRPPRKGNH AERVGAGAPV YPAAVLEHLT AETPEPAGNA ARDNKKTRII PRHLQLAVRN DEELNKLLGG VTIAQGGVPP NIQAVLSPKK TESHKPGKNK

・Histone H2B
MPDPAKSAPA PKKGSKKAVT KVQKKDGKKR KRSRKESYSV YVYKVLKQVH PDTGISSKAM GIMNSFVNDI FERIAGEASR LAHYNKRSTI TSREIQTAVR LLLPGELAKH AVSEGTKAVT KYTSSNPRNL SPTKPGGSED RQPPPSQLSA IPPFCLVLRA GIAGQV

・Histone H3.3
MARTKQTARK STGGKAPRKQ LATKAARKSA PATGGVKKPH RYRPGTVALR EIRRYQKSTE LLIRKLPFQR LVREIAQDFK TDLRFQSSAV MALQEACEAY LVGLFEDTNL CAIHAKRVTI MPKDIQLARR IRGERA

・ Histone H4
SGRGK GGKGL GKGGA KRHRK VLRDN IQGIT KPAIR RLARR GGVKR ISGLI YEETR GVLKV FLENV IRDAV TYTEH AKRKT VTAMD VVYAL KRQGR TLYGF GG

Supplementary Figure 2. Sequence of each purified histone subtype.

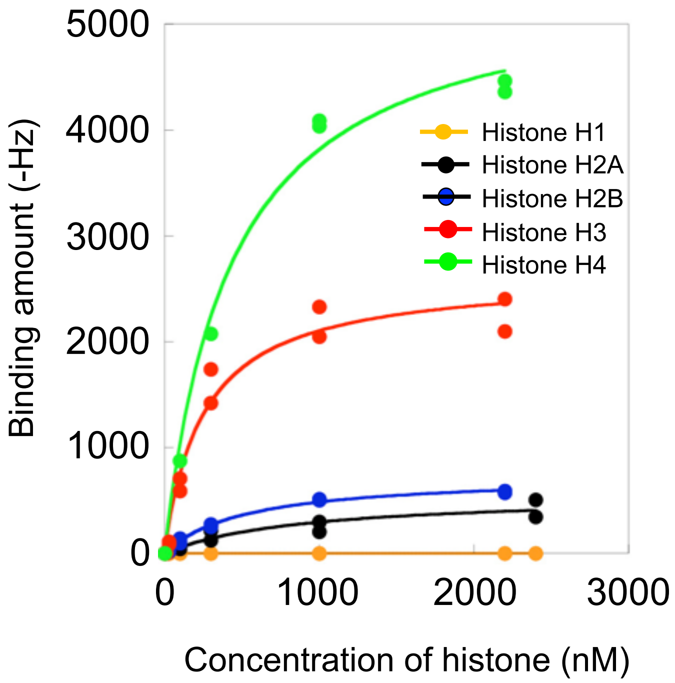

Supplementary Figure 3. Interaction of HNP4 to each histone subtype. The surface of the QCM was functionalized with HNP4 and purified histones were added to the QCM cells. Data represent the means for independent duplicate measurements.

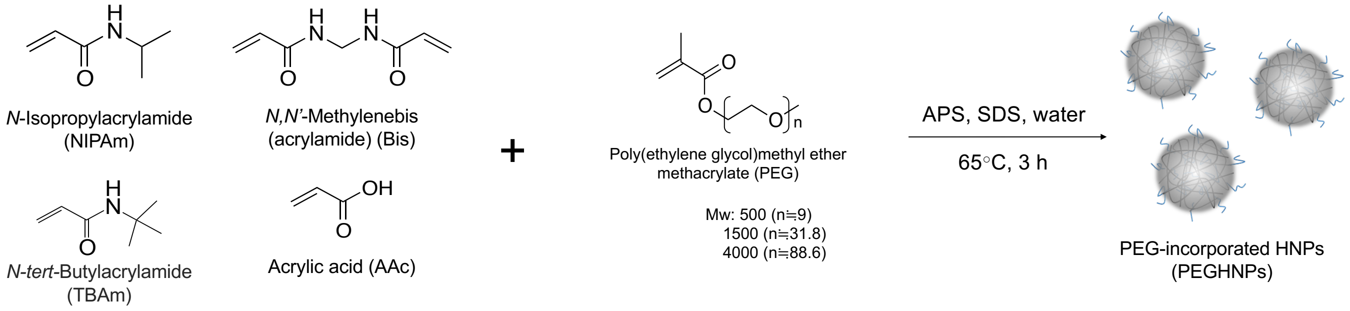

Supplementary Figure 4. Functional monomers used for PEG-incorporated polymer nanoparticles (PEGHNP) synthesis and schematic showing the general synthesis of PEGHNP.

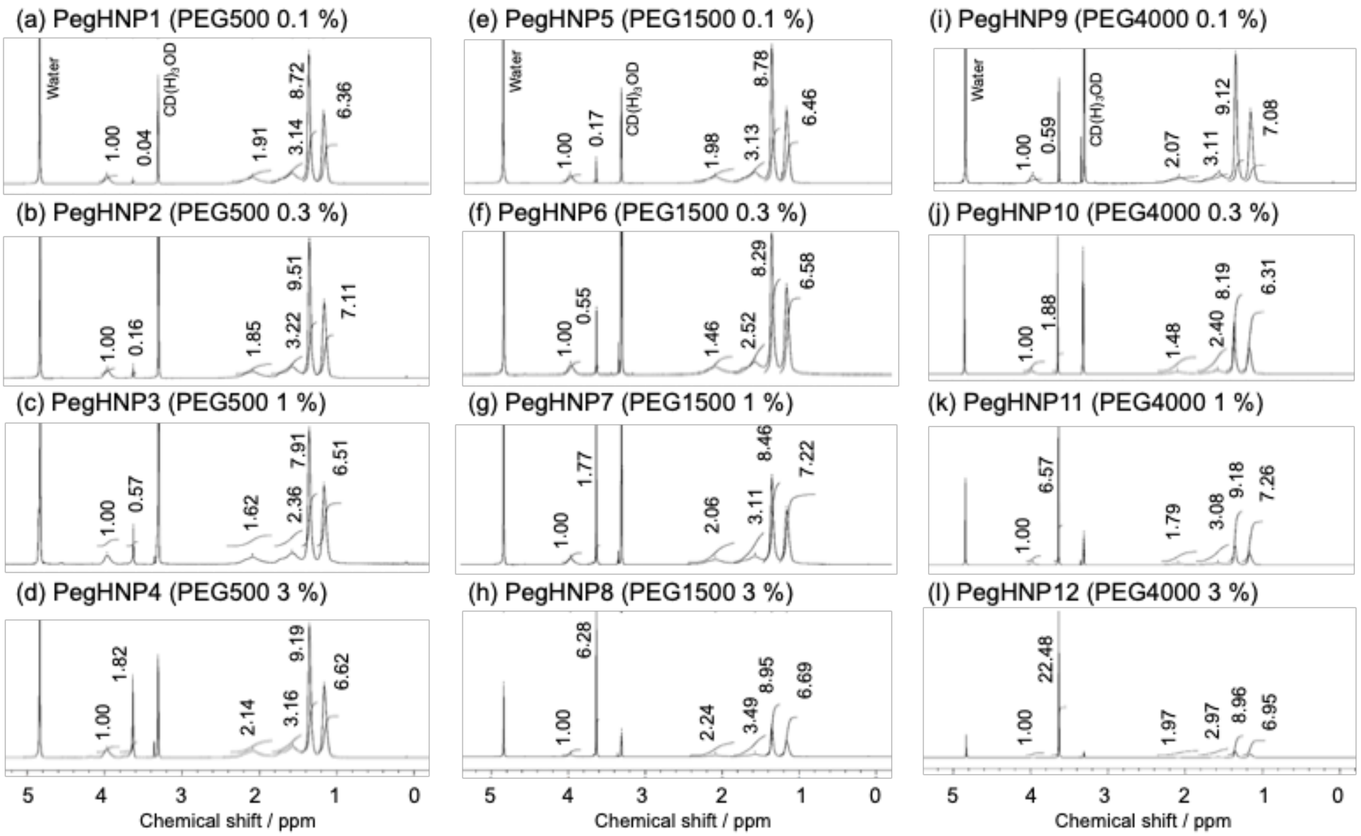

Supplementary Figure 5. NMR spectrum of PEGHNPs.

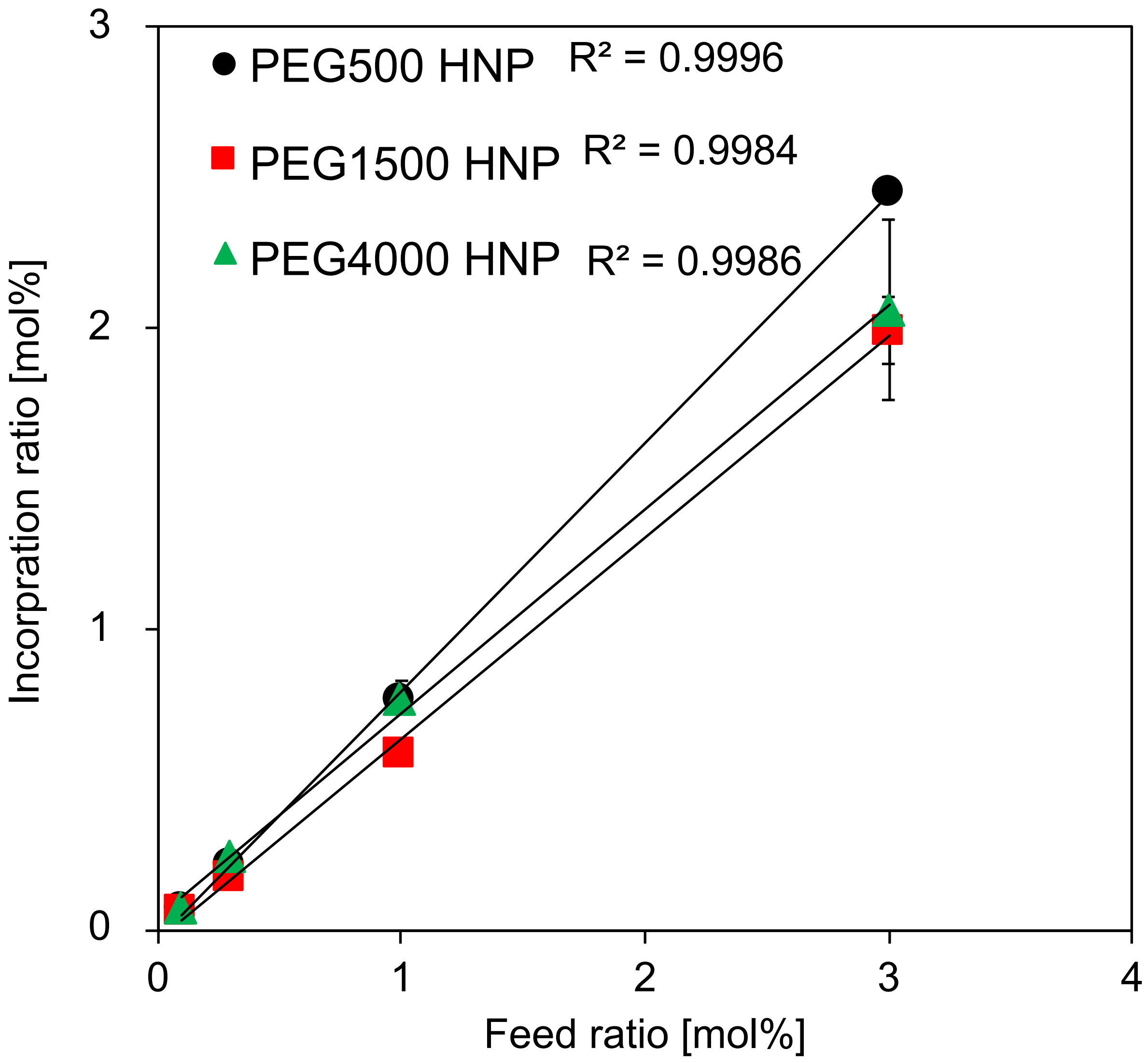

Supplementary Figure 6. Relationship between feed and incorporate percentage of each PEG monomer in PEGHNPs. Incorporation percentage was calculated by ^{1}HNMR. Data represent the means ± s.d. n = 3.

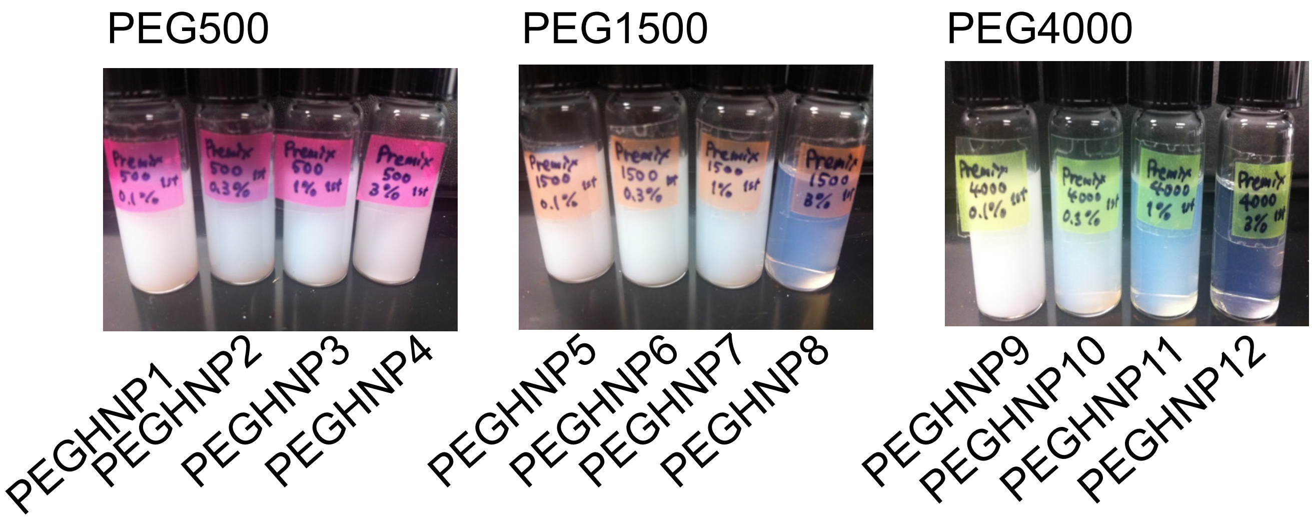

Supplementary Figure 7. Picture of each PEGHNP after the synthesis.

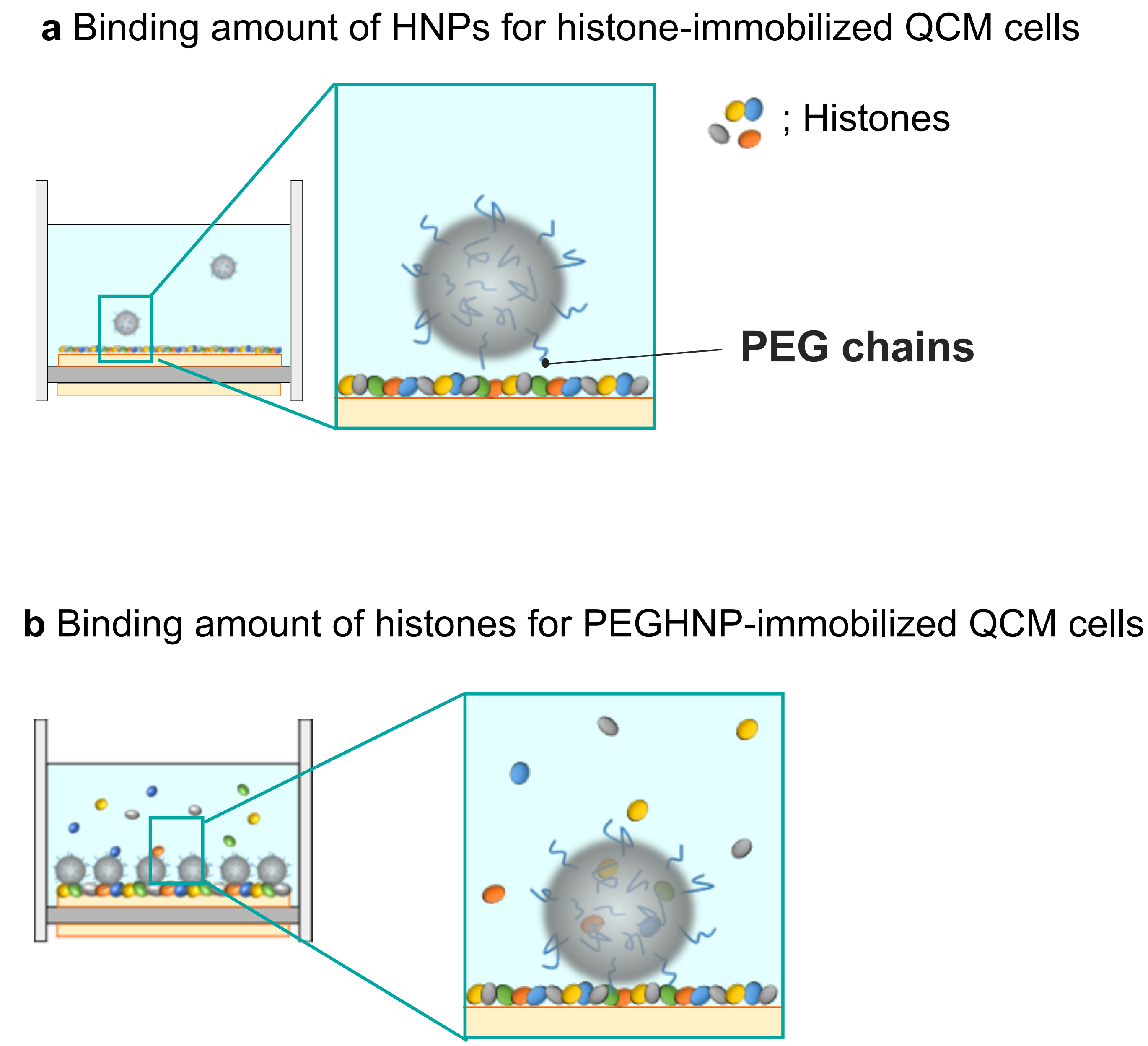

Supplementary Figure 8. Schematic image of QCM analysis. (a) Binding amount of PEGHNPs for histone-immobilized QCM cells. Histones were immobilized onto QCM cells. PEG chains on the HNP surface inhibits binding of HNP to histone-immobilized QCM cells. (b) Binding amount of histones for PEGHNP-immobilized QCM cells. Histones bind to inside of PEGHNP through PEG chains.

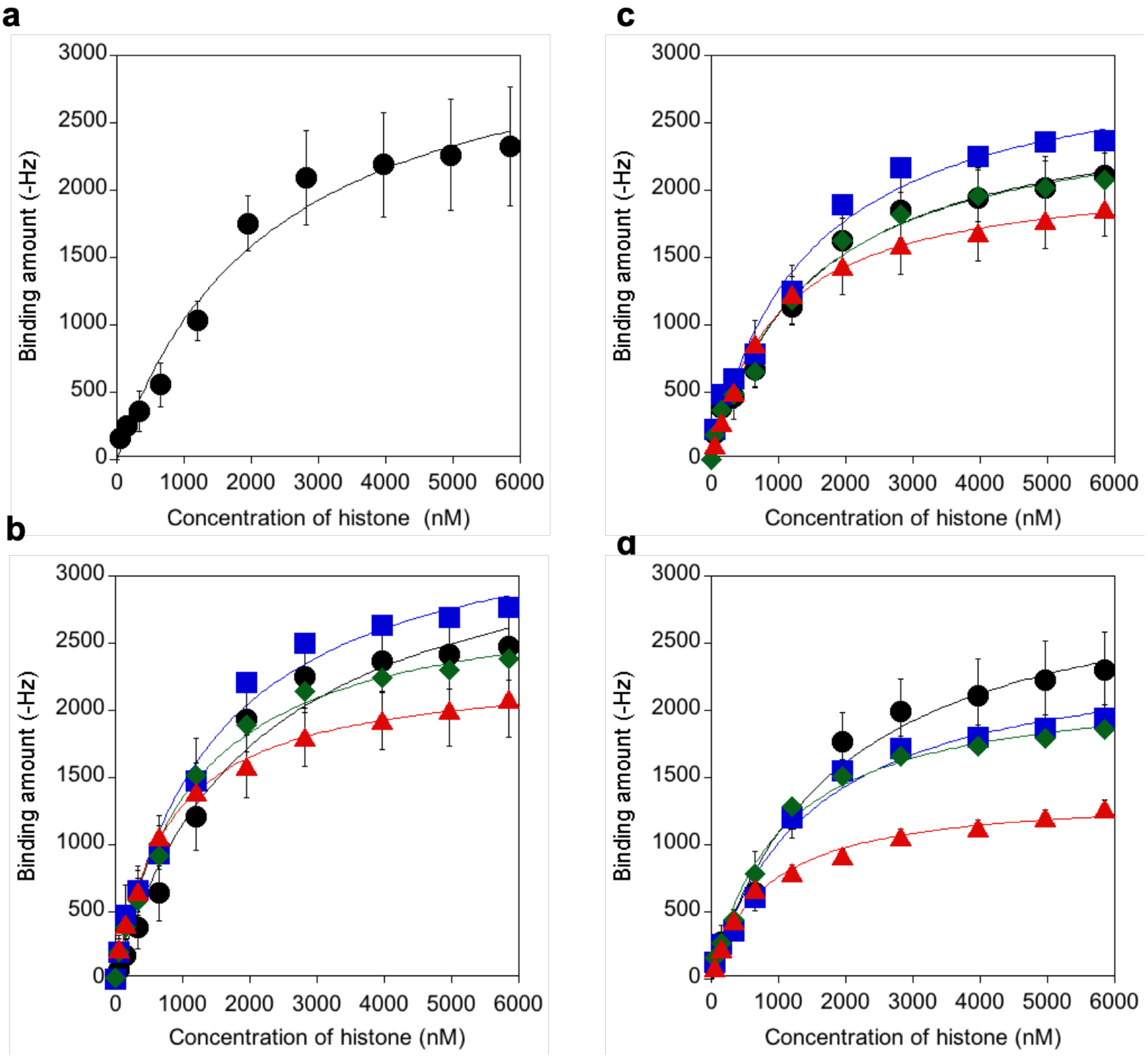

Supplementary Figure 9. QCM analysis of PEGHNPs –histone interaction. The surface of the QCM was functionalized with HNPs and solutions of histones were added to the QCM cells. Binding amount of histone against naked HNPs (a), PEGHNP1-4 (b), PEGHNP5-8 (c) or PEGHNP9-12 (d). (b,c,d) Black; PEGHNP1, 5 and 9, blue; PEGHNP2, 6 and 10, green; PEGHNP3, 7 and 11, red; PEGHNP4, 8 and 12. Data represent the means ± s.d. n = 3.

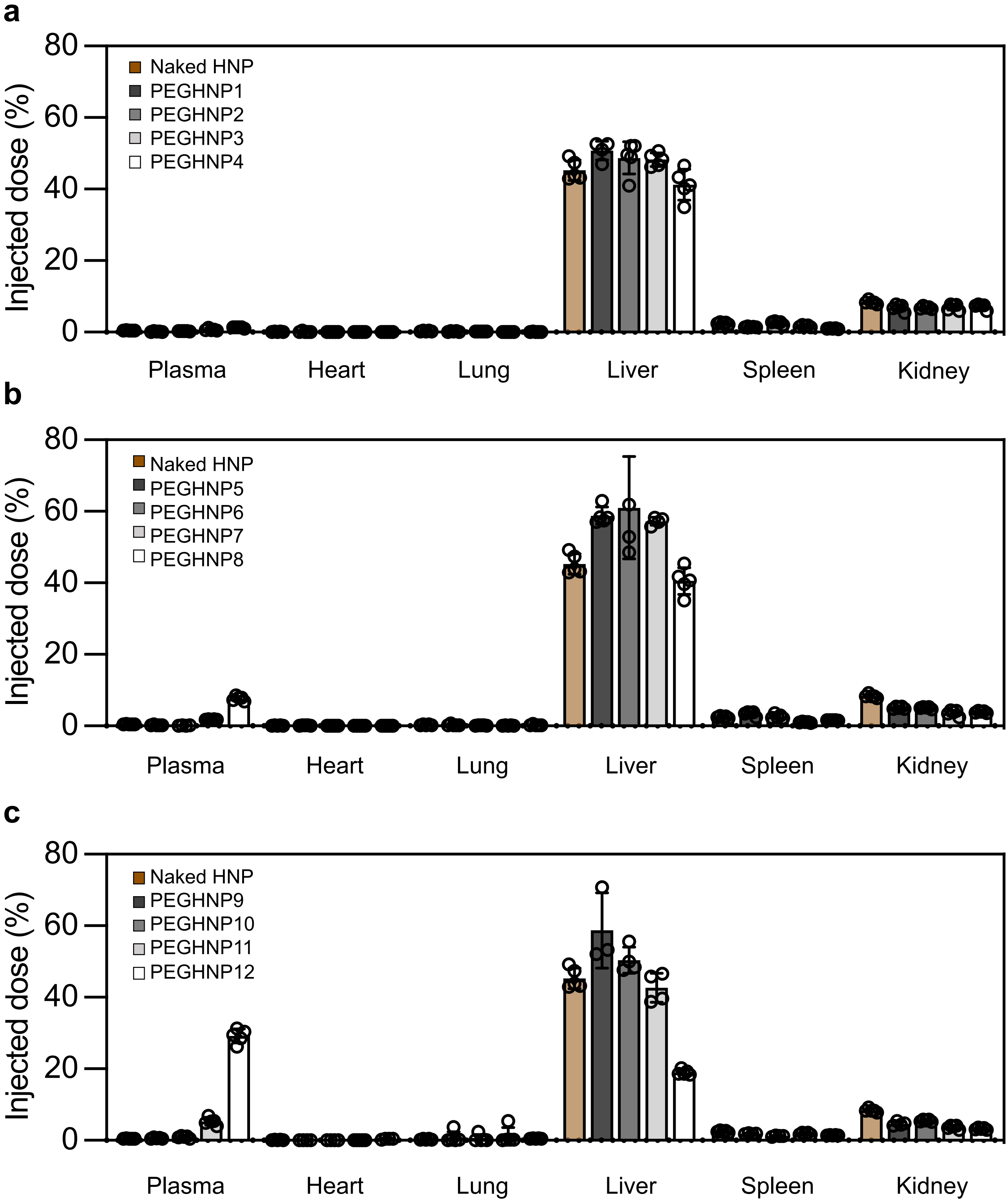

Supplementary Figure 10. Biosdistribution of (a) PEG500, (b) PEG1500, and (c) PEG4000-containing HNPs at 3h after the intravenous injection. Data represent the means ± s.d. n = 5.

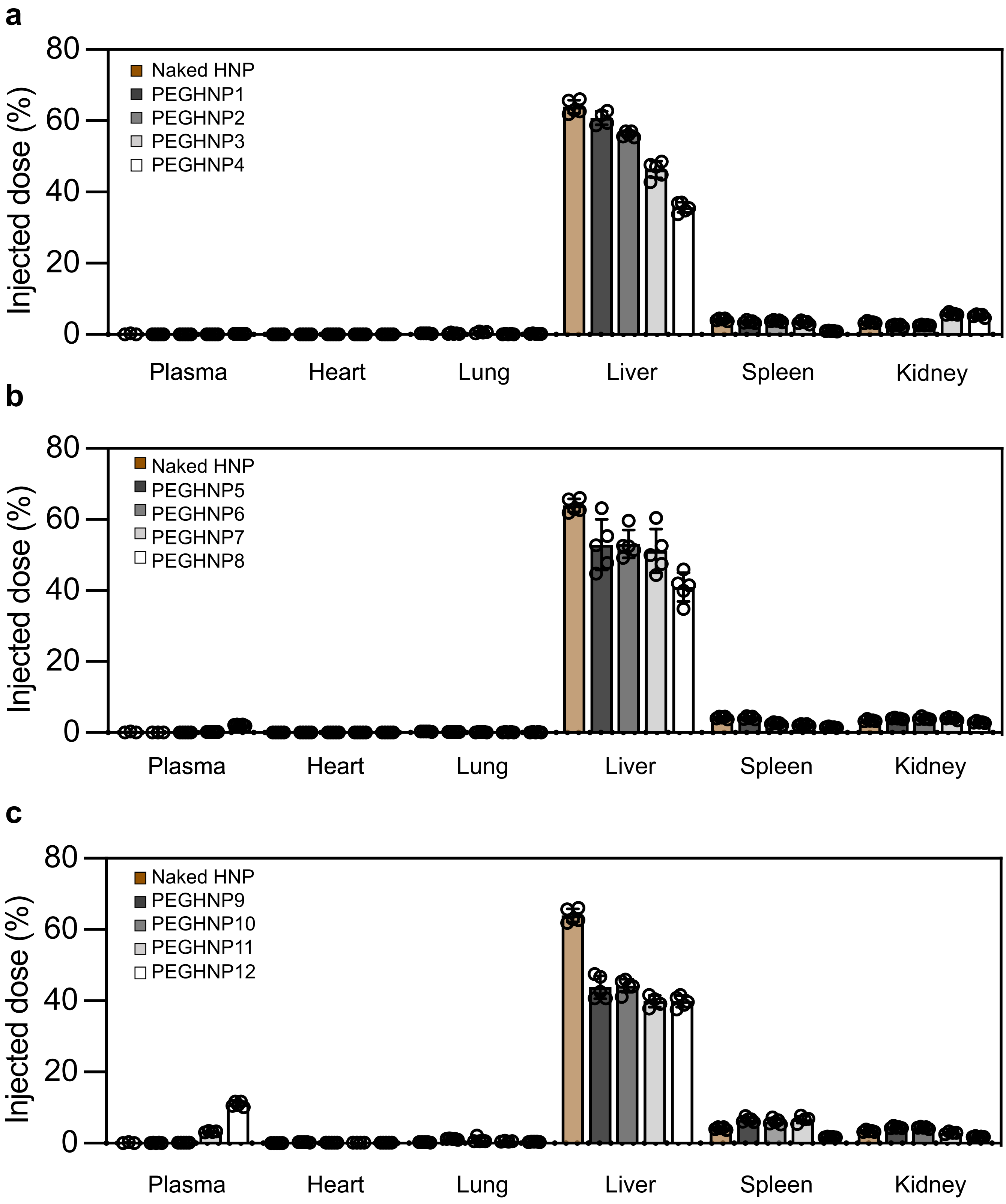

Supplementary Figure 11. Biosdistribution of (a) PEG500, (b) PEG1500, and (c) PEG4000-containing HNPs at 24h after the intravenous injection. Data represent the means ± s.d. n = 5.

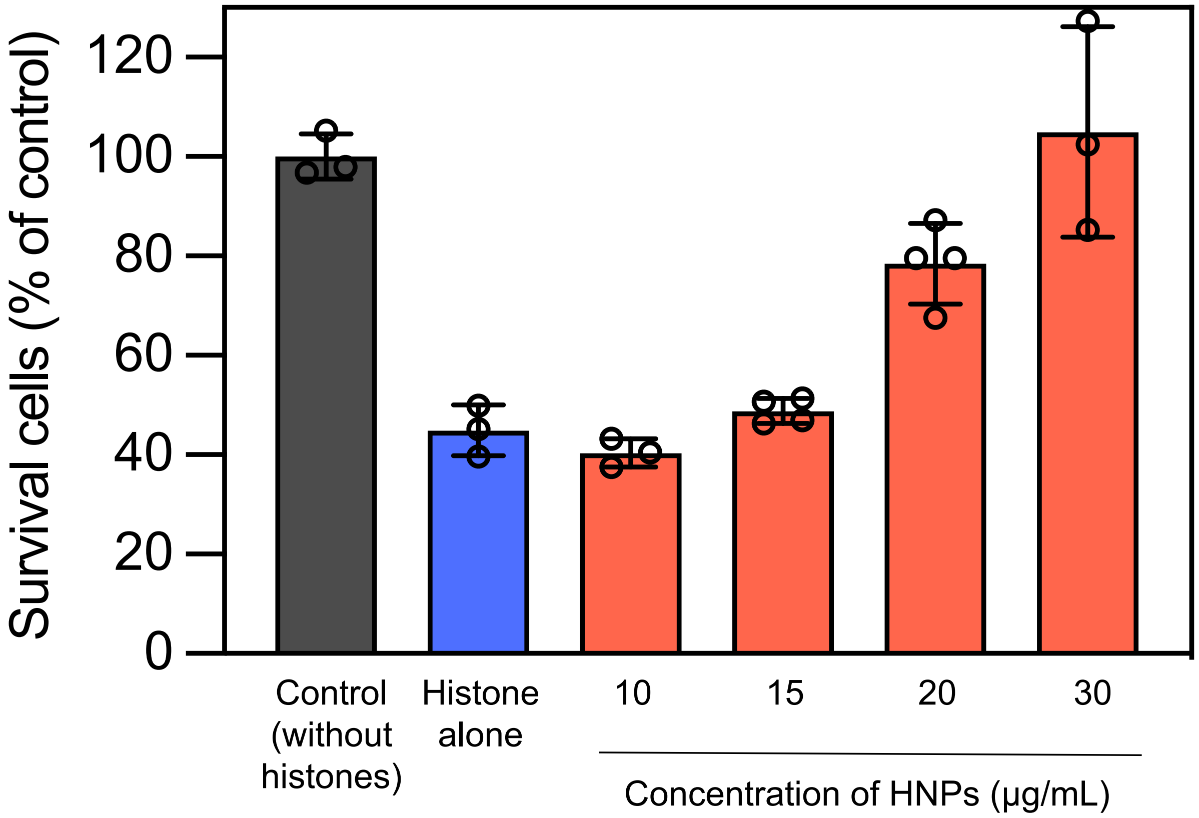

Supplementary Figure 12. Dose-dependent histone toxicity neutralization effect by naked HNPs. Data represent the means ± s.d. n = 3~4.

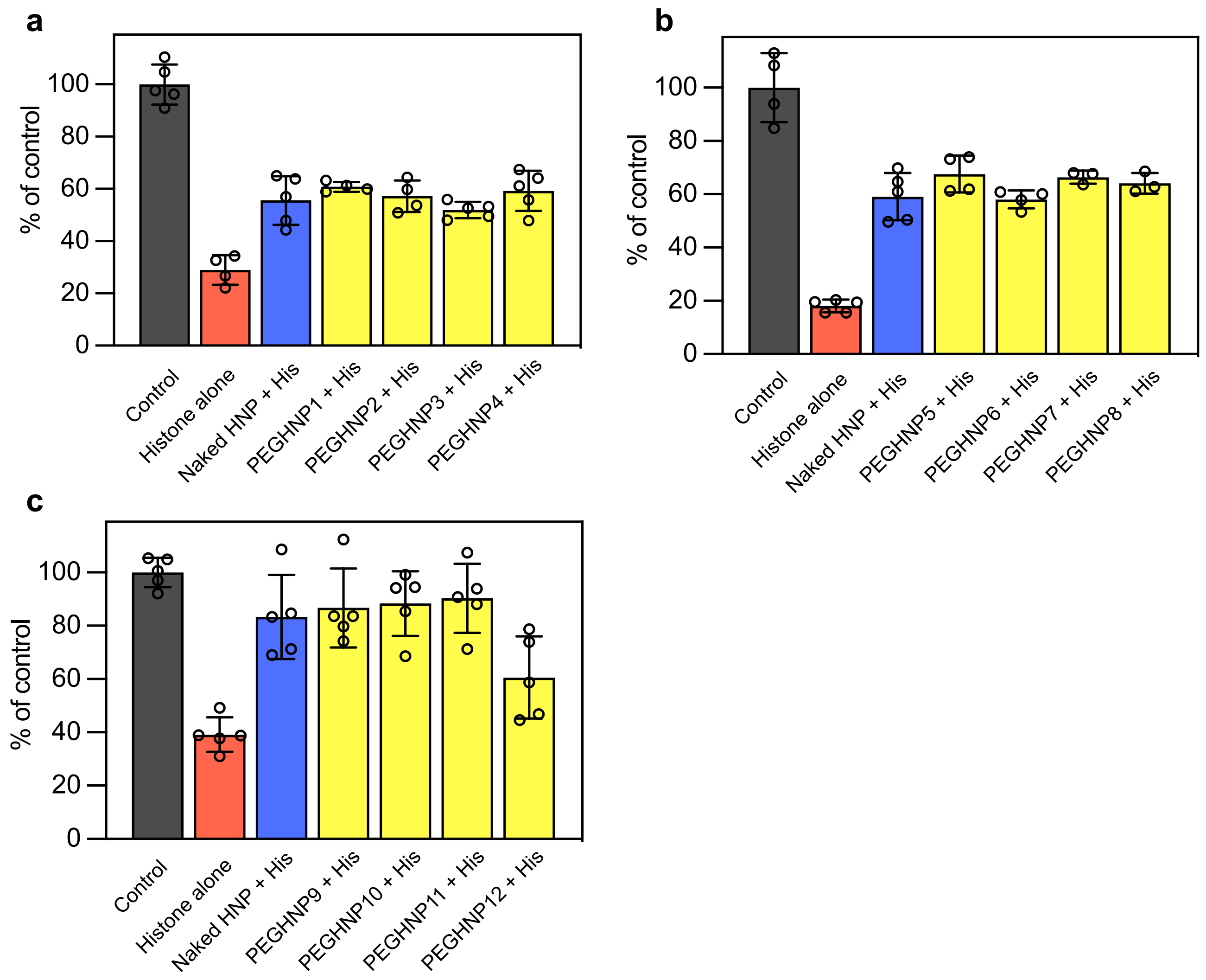

Supplementary Figure 13. Histone toxicity neutralization effect by PEGHNPs. Histone (45 μg/ml) and PEGHNPs (20μg/ml) were incubated for 30 min. Then, the complex was added to the 2H-11 cells. At 24 h after the addition, viable cells were determined by WST-8 assay. (a) PEG500, (b) PEG1500, and (c) PEG4000-containing HNPs. Data represent the means ± s.d. n = 4~5.

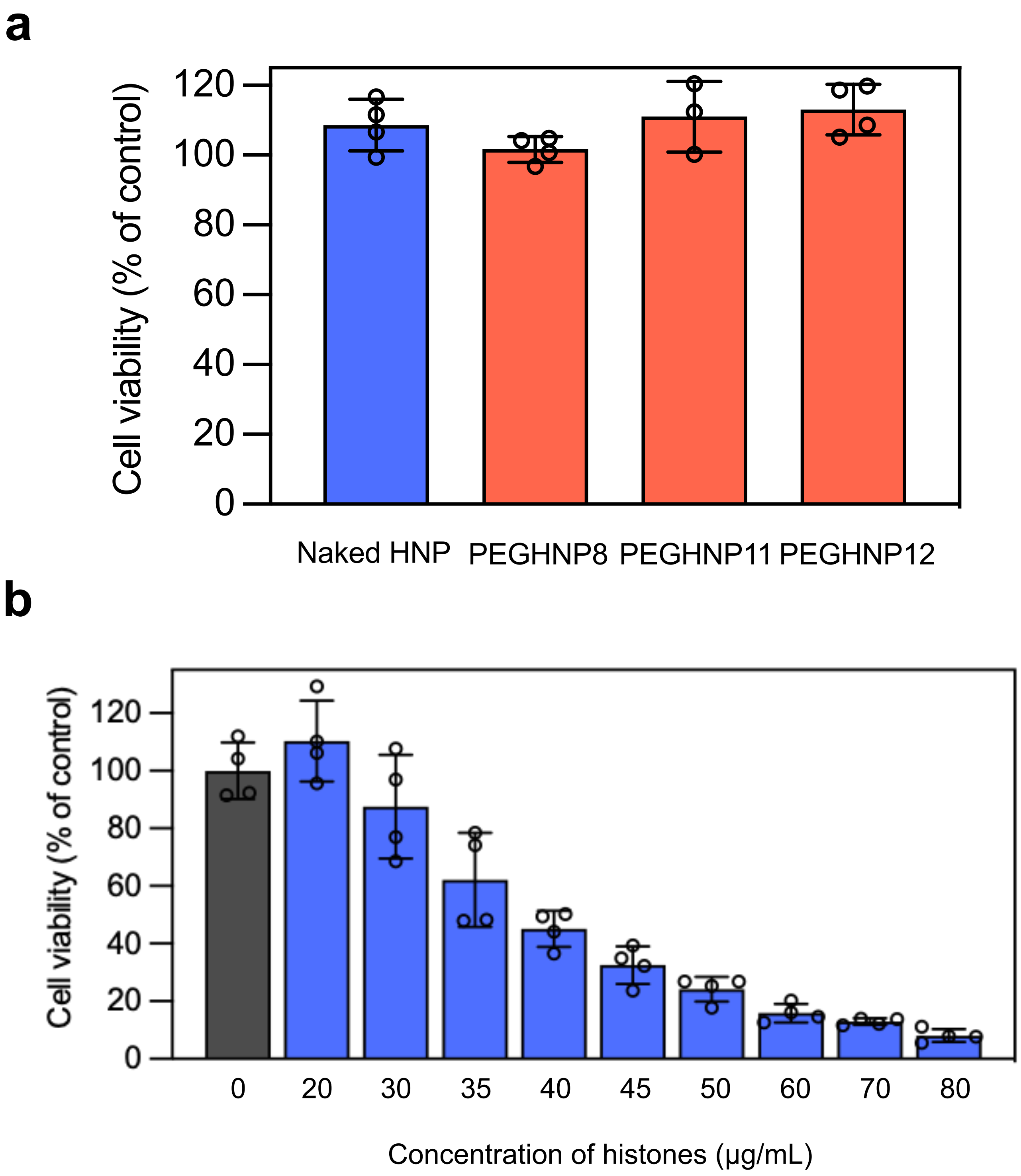

Supplementary Figure 14. Cytotoxicity of (a) PEGHNPs alone and (b) histones alone. 2H-11 cells were incubated with PEGHNPs (100 μg/ml) or histones for 24 min. Then, viable cells were determined by WST-8 assay. Data represent the means ± s.d. n = 3~4.

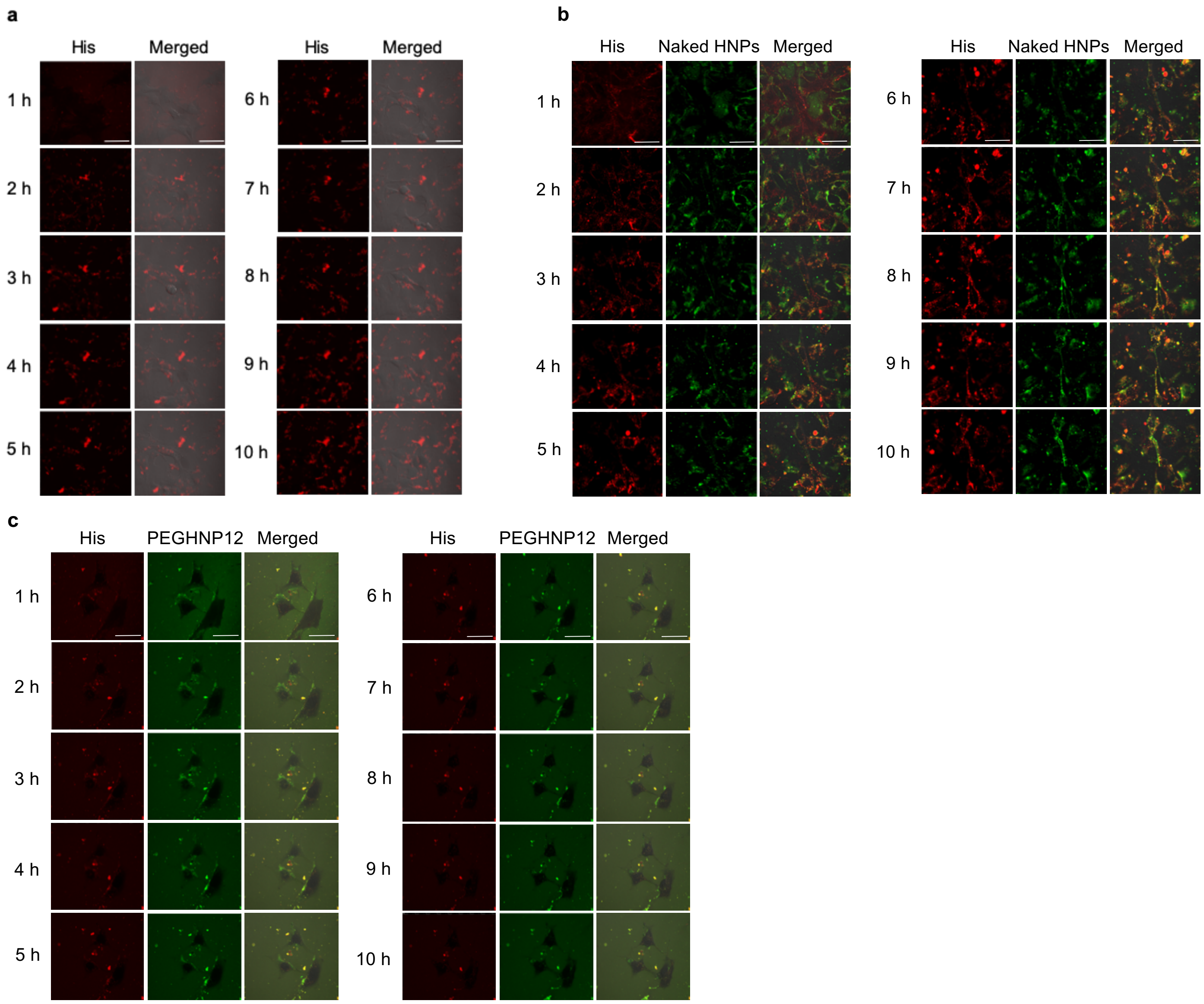

Supplementary Figure 15. Intracellular distribution of Cy5-histone and NPs. 2H-11 cells were added with CY5-histones and FITC-HNPs. Then, intracellular distribution of Cy5-histone and FITC-naked HNPs were monitored for 10 h after the sample addition. a; Histone alone, b; histone + naked HNPs, c, histone + PEGHNP12. Red; histone, Green; HNPs. Bar; 50 µm. Experiment repeated two times and three pictures were took in each experiment.

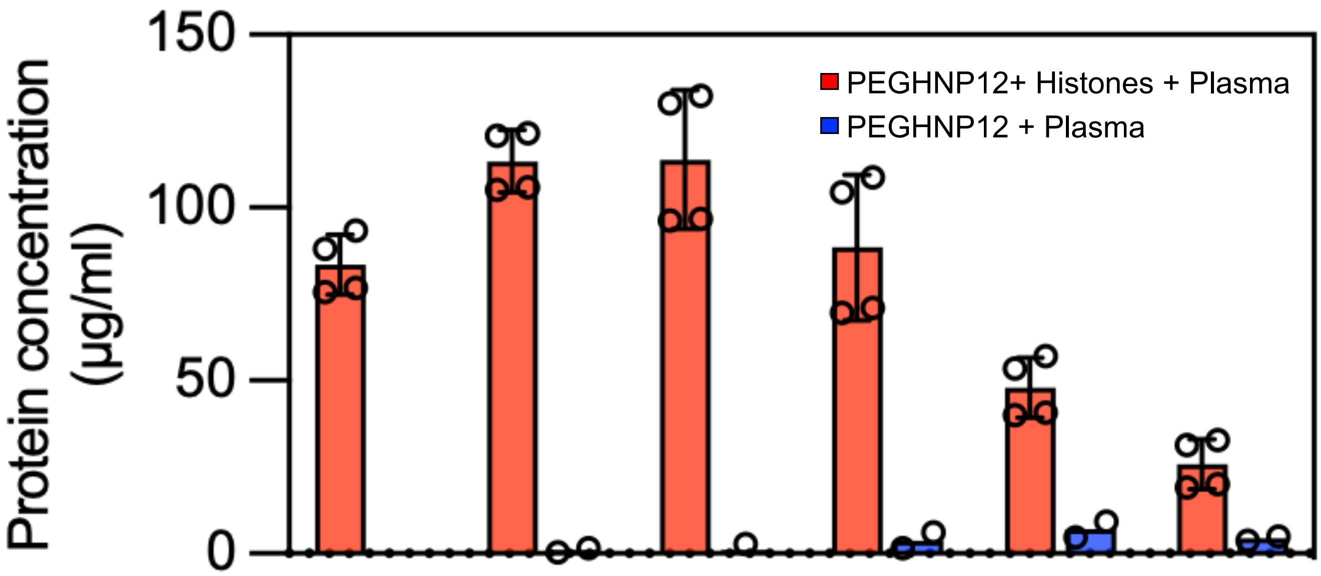

Supplementary Figure 16. Amount of protein in HNP fractions. PEGHNP12 (100 µg/ml) was incubated in 50% plasma with or without histones at 37 °C for 1 hour. Then, PEGHNP12 was purified with gel filtration chromatography. Then, protein amounts in HNP fractions were measured by BCA assay. Data represent the means (± s.d.) n = 2~4.

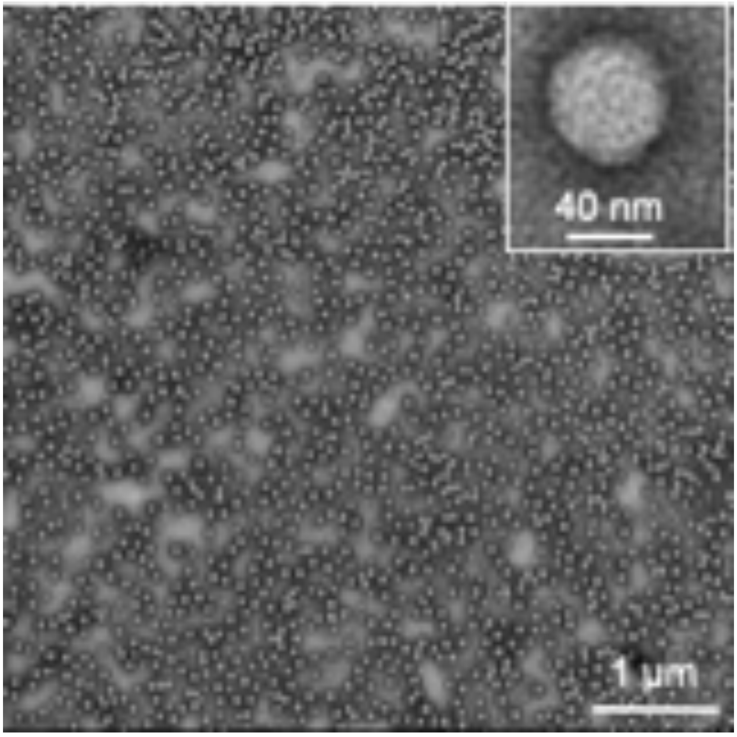

Supplementary Figure 17. TEM image of histone and PEGHNP12 complex. Experiment repeated two times and five pictures were took in each experiment.

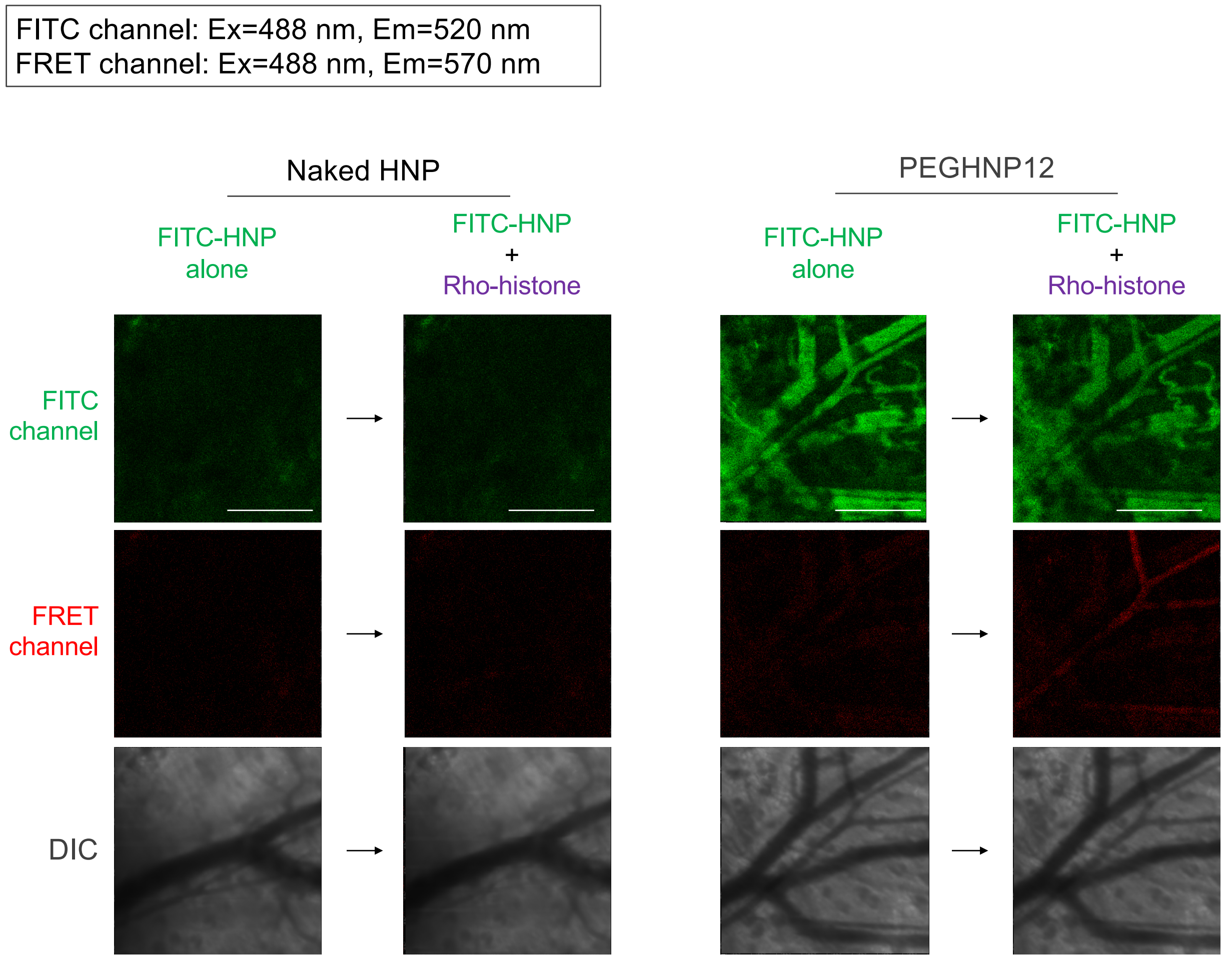

Supplementary Figure 18. Observation of HNP and histone interaction in the bloodstream (ear) by confocal laser-scanning microscopy. The FITC-HNP and FRET channels were recorded for 10 min before and after the Rho-histone injection. Bar; 0.5 mm. Experiment repeated two times and three pictures were took in each experiment.

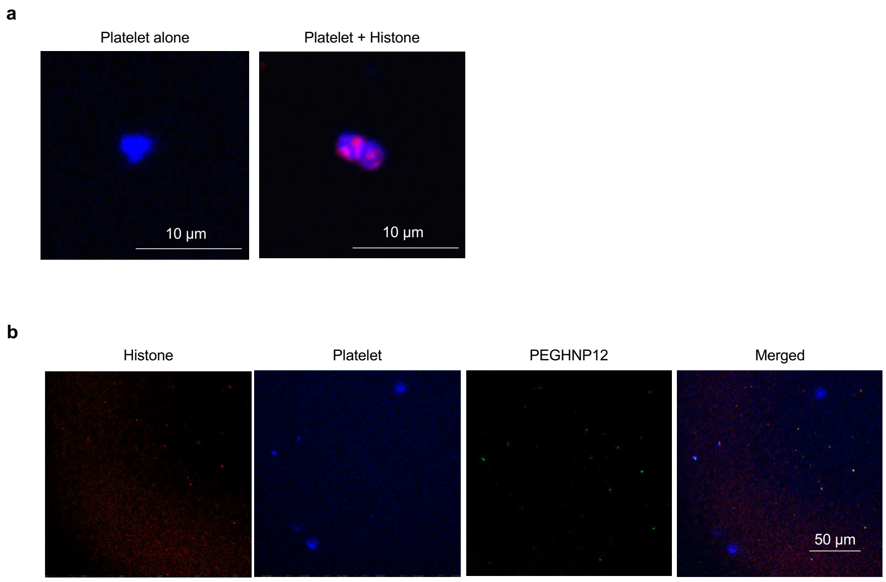

Supplementary Figure 19. Inhibition of histone and platelets interaction by PEGHNP12. Platelets (1 x 10^7 cells) were incubated with Cy5-histone (1 mg/ml) and/or FITC-PEGHNP12 (1 mg/ml). (a) Platelets (blue) with Cy5 histone (red). (b) Platelets (blue) with Cy5 histone (red) and PEGHNP12 (green). Experiment repeated two times and five pictures were took in each experiment.

Supplementary Table 1. NIPAm, PEG and TBAm incorporation ratio in each PEGHNPs.

Feed ratio of PEG monomer	Entry	PEG M.W.	Experimental Incorporation ratio (mol%)		
			NIPAm (53 mol%)	TBAm (40 mol%)	PEG
0.1 mol%	PEGHNP1	500	53	43.8±1.5	0.07±0.02
	PEGHNP5	1500	53	44.1±2.3	0.06±0.01
	PEGHNP9	4000	53	47.3±0.2	0.07±0.01
0.3 mol%	PEGHNP2	500	53	43.1±3.6	0.22±0.03
	PEGHNP6	1500	53	40.9±2.6	0.17±0.01
	PEGHNP10	4000	53	46.4±0.7	0.24±0.02
1 mol%	PEGHNP3	500	53	43.3±3.0	0.77±0.04
	PEGHNP7	1500	53	42.7±1.8	0.59±0.04
	PEGHNP11	4000	53	43.9±3.6	0.77±0.06
3 mol%	PegHNP4	500	53	44.1±2.9	2.46±0.04
	PEGHNP8	1500	53	43.3±3.9	1.99±0.11
	PEGHNP12	4000	53	40.4±0.3	2.06±0.30

Supplementary Table 2. Binding affinity (K_d) of HNPs against histone mixture.

Feed ratio of PEG monomer	Entry	PEG M.W.	Experimental Incorporation ratio (mol%)		
			NIPAm (53 mol%)	TBAm (40 mol%)	PEG
0.1 mol%	PEGHNP1	500	53	43.8±1.5	0.07±0.02
	PEGHNP5	1500	53	44.1±2.3	0.06±0.01
	PEGHNP9	4000	53	47.3±0.2	0.07±0.01
0.3 mol%	PEGHNP2	500	53	43.1±3.6	0.22±0.03
	PEGHNP6	1500	53	40.9±2.6	0.17±0.01
	PEGHNP10	4000	53	46.4±0.7	0.24±0.02
1 mol%	PEGHNP3	500	53	43.3±3.0	0.77±0.04
	PEGHNP7	1500	53	42.7±1.8	0.59±0.04
	PEGHNP11	4000	53	43.9±3.6	0.77±0.06
3 mol%	PegHNP4	500	53	44.1±2.9	2.46±0.04
	PEGHNP8	1500	53	43.3±3.9	1.99±0.11
	PEGHNP12	4000	53	40.4±0.3	2.06±0.30

Supplementary Table 3. HNP sizes in PBS, histones or 50% plasma solution.

	in PBS	with histones	with plasma
Naked HNP	120 ± 2	4670 ± 790	128 ± 3
PEGHNP12	45 ± 1	54 ± 3	41 ± 1
			Size (d.nm)
