# Supplementary material for: Synthetic hydrogel nanoparticles for sepsis therapy
Source: Nat Commun. 2021 Sep 21;12:5552. doi: 10.1038/s41467-021-25847-2 (PMC8455629; doi:10.1038/s41467-021-25847-2)
Supplement: Supplementary file 2 — Reporting Summary [file 41467_2021_25847_MOESM2_ESM.pdf]

nature research

Corresponding author(s): Kenneth J. Shea

Last updated by author(s): Aug 20, 2021

Reporting Summary

Nature Research wishes to improve the reproducibility of the work that we publish. This form provides structure for consistency and transparency in reporting. For further information on Nature Research policies, see our Editorial Policies and the Editorial Policy Checklist.

Statistics

For all statistical analyses, confirm that the following items are present in the figure legend, table legend, main text, or Methods section.

n/a	Confirmed	
☐	☒	The exact sample size (*n*) for each experimental group/condition, given as a discrete number and unit of measurement
☐	☒	A statement on whether measurements were taken from distinct samples or whether the same sample was measured repeatedly
☐	☒	The statistical test(s) used AND whether they are one- or two-sided<br>*Only common tests should be described solely by name; describe more complex techniques in the Methods section.*
☐	☒	A description of all covariates tested
☒	☐	A description of any assumptions or corrections, such as tests of normality and adjustment for multiple comparisons
☐	☒	A full description of the statistical parameters including central tendency (e.g. means) or other basic estimates (e.g. regression coefficient) AND variation (e.g. standard deviation) or associated estimates of uncertainty (e.g. confidence intervals)
☒	☐	For null hypothesis testing, the test statistic (e.g. *F*, *t*, *r*) with confidence intervals, effect sizes, degrees of freedom and *P* value noted<br>*Give P values as exact values whenever suitable.*
☒	☐	For Bayesian analysis, information on the choice of priors and Markov chain Monte Carlo settings
☒	☐	For hierarchical and complex designs, identification of the appropriate level for tests and full reporting of outcomes
☒	☐	Estimates of effect sizes (e.g. Cohen's *d*, Pearson's *r*), indicating how they were calculated

Our web collection on statistics for biologists contains articles on many of the points above.

Software and code

Policy information about availability of computer code

Data collection	Dynamic-light-scattering data was collected by using Malvern Zetasizer Nano software version 2.2.Absorbance was measured with an Infinite® M200 (Tecan Group, Männedorf, Switzerland). ex vivo imaging of blood and each organ was performed with an in vivo imaging system (Xenogen IVIS Lumina System) coupled to the Living Image software for data acquisition (Living Image 3.2, Xenogen Corp., Alameda, CA, USA).
Data analysis	Kaleidagraph (Version 4.5.3) and Prism 9 (Version 9.2.0) was used for the data analysis.

For manuscripts utilizing custom algorithms or software that are central to the research but not yet described in published literature, software must be made available to editors and reviewers. We strongly encourage code deposition in a community repository (e.g. GitHub). See the Nature Research guidelines for submitting code & software for further information.

Data

Policy information about availability of data

All manuscripts must include a data availability statement. This statement should provide the following information, where applicable:

- Accession codes, unique identifiers, or web links for publicly available datasets
- A list of figures that have associated raw data
- A description of any restrictions on data availability

No datasets were generated or analyzed during the current study.

Field-specific reporting

Please select the one below that is the best fit for your research. If you are not sure, read the appropriate sections before making your selection.

- [x] Life sciences
- [ ] Behavioural & social sciences
- [ ] Ecological, evolutionary & environmental sciences

For a reference copy of the document with all sections, see nature.com/documents/nr-reporting-summary-flat.pdf

Life sciences study design

All studies must disclose on these points even when the disclosure is negative.

Sample size	In mice, sample size of n=5 or 6 for the in vivo neutraliaztion and biodistribution studies was determined. In all experiments, the sample sizes were determined by estimating the minimum number of samples to obtain a statistically significant difference.
Data exclusions	No data were excluded from analysis.
Replication	in vivo neutraliaztion and biodistribution studies were was performed once with 6 or more animals in each group. For all experiments, 2 independent experiments were performed. In addition, all attempts for replication were successfull.
Randomization	Animals were randomly assigned to the treatment conditions by the animal technician before the experiment started. For in vitro experiments, all experiments allocated random by who was not involved in the experiment thereafter
Blinding	For animal experiments, data collection and analysis were performed blinded.<br>For in vitro experiments, all analyses of cytotoxicity, cellular uptake, and localization of HNP and histones were done fully blinded. Labeling of sections was done by numbering at the time of sectioning.

Reporting for specific materials, systems and methods

We require information from authors about some types of materials, experimental systems and methods used in many studies. Here, indicate whether each material, system or method listed is relevant to your study. If you are not sure if a list item applies to your research, read the appropriate section before selecting a response.

Materials & experimental systems

n/a	Involved in the study
[ ]	[x] Antibodies
[ ]	[x] Eukaryotic cell lines
[x]	[ ] Palaeontology and archaeology
[ ]	[x] Animals and other organisms
[x]	[ ] Human research participants
[x]	[ ] Clinical data
[x]	[ ] Dual use research of concern

Methods

n/a	Involved in the study
[x]	[ ] ChIP-seq
[x]	[ ] Flow cytometry
[x]	[ ] MRI-based neuroimaging

Antibodies

Antibodies used	mouse integrin alpha 2b/CD41 alexa fluor® 594-conjugated antibody (FAB41181T-100UG) was purchased from R&D systems (USA, Minneapolis, MN) and 25 times dilute for use.
Validation	mouse integrin alpha 2b/CD41 alexa fluor® 594-conjugated antibody; mouse species reactivity; Flow Cytometry applicable.

Eukaryotic cell lines

Policy information about cell lines

Cell line source(s)	2H-11 murine endothelial cells was purchased from ATCC (Virginia, USA)
Authentication	2H-11 cell was authenticated by STR-PCR.
Mycoplasma contamination	2H-11 was confirmed as mycoplasma negative by direct DNA staining with Hoescht 33342.
Commonly misidentified lines (See ICLAC register)	No commonly misidentified cell lines were used.

Animals and other organisms

Policy information about studies involving animals; ARRIVE guidelines recommended for reporting animal research

Laboratory animals	Five-week-old BALB/c male mice were purchased from Japan SLC Inc. (Shizuoka, Japan)
Wild animals	The study did not use wild animals.
Field-collected samples	The study did not involve samples collected from the field
Ethics oversight	The animals were cared for according to the Animal Facility Guidelines of the University of Shizuoka. All animal experiments were approved by the Animal and Ethics Review Committee of the University of Shizuoka.

Note that full information on the approval of the study protocol must also be provided in the manuscript.
